# Controlling exposure to As and Cd from rice via irrigation management

**DOI:** 10.1007/s10653-024-02116-x

**Published:** 2024-07-29

**Authors:** Matt A. Limmer, Angelia L. Seyfferth

**Affiliations:** Department of Plant and Soil Science, University of Delaware, Newark, DE USA

**Keywords:** Water management, Aerobic rice, Alternate wetting and drying, Trace metals, Geohealth

## Abstract

Irrigation management controls biogeochemical cycles in rice production. Under flooded paddy conditions, arsenic becomes plant-available as iron-reducing conditions ensue, while oxic conditions lead to increased plant availability of Cd in acidic soils. Because Cd enters rice through Mn transporters, we hypothesized that irrigation resulting in intermediate redox could simultaneously limit both As and Cd in rice grain due to As retention in soil and Mn competition for Cd uptake. In a 2 year field study, we used 6 irrigation managements that varied in extent and frequency of inundation, and we observed strong effects of irrigation management on porewater chemistry, soil redox potentials, plant As and Cd concentrations, plant nutrient concentrations, and methane emissions. Plant As decreased with drier irrigation management, but in the grain this effect was stronger for organic As than for inorganic As. Grain organic As, but not inorganic As, was strongly and positively correlated with cumulative methane emissions. Conversely, plant Cd increased under more aerobic irrigation management and grain Cd was negatively correlated with porewater Mn. A hazard index approach showed that in the tested soil with low levels of As and Cd (5.4 and 0.072 mg/kg, respectively), irrigation management could not simultaneously decrease grain As and Cd. Many soil properties, such as reducible As, available Cd, soil pH, available S, and soil organic matter should be considered when attempting to optimize irrigation management when the goal is decreasing the risk of As and Cd in rice grain.

**Supplementary Information:**

The online version contains supplementary material available at 10.1007/s10653-024-02116-x.

## Introduction

Irrigation management in rice differs from that in other crops because rice thrives under flooded soil conditions. While rice can be grown under completely aerobic conditions (i.e., nonflooded), a flood is often used for weed control. The flood duration can vary due to weather, water availability, farmer preferences, the need to drive machinery on the field, insect and disease control, nutrient management, and historical practices, but a flood is typically maintained throughout most of the growing season (Bouman et al., 2007a, b). The flood may be interrupted one or more times throughout the growing season, allowing the water to drain to a predetermined water level below the ground surface or soil moisture content, a process called a mid-season drain or alternate wetting and drying (AWD). Changes in irrigation management to more aerobic conditions (i.e., less flooded) have often been driven by declining water availability either due to groundwater depletion or drought (Bouman, Lampayan, et al., 2007).

Irrigation management controls the biogeochemical cycles and availability of several elements relevant to rice production. When rice paddies are flooded, oxygen is rapidly depleted and alternative electron acceptors are used by microorganisms for respiration. Nitrate is reduced to ammonium, manganese and iron oxides are reduced to their divalent, mobile forms, sulfate is reduced to sulfide, and methane is produced from more oxidized forms of carbon. Of these alternative electron acceptors, manganese, iron, and carbon are the most prevalent and consequential in many rice soils. These processes interact with important contaminants in rice production. Under iron-reducing conditions, arsenic-bearing iron (oxyhydr)oxide minerals are reductively dissolved, and arsenate is reduced to arsenite, increasing arsenic (As) availability to rice and enabling rice accumulation of this carcinogen (Bose & Sharma, 2002). Conversely, cadmium (Cd) forms the sparingly soluble CdS compound under sulfate-reducing conditions (Fulda et al., 2013), which is one of several processes that drastically limit the availability of Cd to rice under flooded conditions. Flooded conditions also result in the production of methane (CH_4_), and rice production is responsible for ~ 10% of anthropogenic CH_4_ emissions (IPCC, 2013). Thus, changes in irrigation management can affect nutrient availability, contaminant availability, and greenhouse gas emissions with consequent impacts on human health and global climate.

The contrasting effects of irrigation management on As and Cd mobility in soil have led some to parameterize tradeoff curves for As and Cd as a function of soil redox potential with the ultimate goal of finding irrigation managements that simultaneously minimize grain As and Cd. Honma et al., (2016) first described such a tradeoff model, where the minimum amount of Cd and As in the porewater, relative to their respective maximums, occurred at a soil redox potential of −73 mV. Using a pot study, Yao et al., (2022) found bioavailable soil As and Cd were minimized at a soil redox potential of −130 mV. In another pot study, Linam et al., (2022) found grain As and Cd were simultaneously minimized at a porewater redox potential near 250 mV. The variability in the reported optimal tradeoff values is concerning, although variability may arise from differences in soil properties, the different dependent variables measured, and redox measurements reported relative to different reference electrodes (i.e., the standard hydrogen electrode, Ag/AgCl, etc.). The redox potentials also vary substantially in time, so temporally averaged data are often reported, which hinders the utility of redox measurements in real-time irrigation management decisions. Additionally, the cost of redox probes and data loggers may preclude widespread adoption by farmers.

The soil redox potential is not a direct driver of As and Cd availability. For As, reductive dissolution of As-containing iron (oxyhydr)oxides and reduction of arsenate to arsenite are the primary drivers of As availability under flooded conditions (Pedersen et al., 2006; Tufano & Fendorf, 2008). The mobilized arsenite in the porewater is readily taken up by the efficient Si transporter system in rice roots (Ma et al., 2008; Seyfferth et al., 2018). Cadmium accumulation in rice is under more complex control. Under sulfate-reducing conditions, the practically insoluble CdS limits Cd availability, but draining paddy soil can oxidize the CdS resulting in available Cd(II) (Fulda et al., 2013). The rate of CdS oxidation depends on the relative concentration of other metal sulfides, such as FeS, MnS, CuS, and ZnS (Barrett & McBride, 2007; Huang, Chen, et al., 2021a, b, c; H. Huang, Ji, et al., 2021a, b, c). Cd bioavailability is also controlled by the soil pH, with Cd adsorption increasing as the pH increases. In well-weathered, acid soils, flooding typically raises the porewater pH to near neutral, due to proton consumption during Fe(III) reduction, while draining soils lowers the porewater pH (Wang et al., 2019). Flooding and draining also affect soil organic matter, which strongly interacts with Cd (Yang et al., 2021). But once available to the plant, Cd is primarily taken up by a root Mn transporter (Sasaki et al., 2012). Collectively, while As availability is enhanced under Fe-reducing conditions, it is unclear whether Mn-reducing conditions would still be sufficiently-reducing to simultaneously limit the mobility of Cd, provide elevated porewater Mn, and avoid the release of As.

The goal of this work was to carefully manipulate redox through varying irrigation managements and use these data to understand the corresponding minimization of health impacts using a hazard index approach. We tested 6 different irrigation managements under field conditions over 2 years and monitored porewater chemistry, plant chemistry, soil redox, and methane emissions. We hypothesized that maintaining a rice paddy system predominately under Mn-reducing conditions but avoiding Fe-reducing conditions could simultaneously minimize grain As and Cd. We also hypothesized that methane emissions and grain As would be strongly correlated.

## Materials and methods

### Experimental design

Irrigation management was varied in 18 paddy mesocosms at the University of Delaware’s Rice Investigation, Communication, and Education (RICE) Facility. Each paddy was 2 × 2 m and contained 0.5 m of soil underlain with an impermeable membrane. The soil, an Ultisol, had a pH of 5.9 and 2.0% soil organic matter as measured by loss on ignition. The soil texture was 28% sand, 45% silt, and 27% clay, classifying the soil as a clay loam. The soil contained 5.4 mg/kg As and 0.072 mg/kg Cd; additional total and extractable elements are shown in Table S1. The reducible Mn in the soil was 180 mg/kg measured using 0.1 M hydroxylamine hydrochloride and 0.01 M nitric acid (Gambrell, 1996). Soil Fe was measured using a sequential extraction with acid ammonium oxalate in the dark and acid ammonium oxalate with ascorbic acid (96 °C) (Wenzel et al., 2001). Using this extraction, the soil contained 2.2 g/kg short-range ordered iron oxides and 12.5 g/kg crystalline iron oxides. Rice had been grown in the paddies for the previous 5–6 years (Limmer & Seyfferth, 2021; Limmer et al., 2018). Twelve of the paddies had been in rice cultivation for 6 years while 6 of the paddies had been in rice cultivation for 5 years. Prior to beginning the experiment, the top 15 cm of soil were removed from each paddy, mixed, and then returned to each paddy to homogenize the soil.

Hybrid rice (*Oryza sativa* ‘CLXL745’) was grown in the paddies for both years of the experiment. Seeds were initially started in the greenhouse and transplanted at the 3–4 leaf stage. Each paddy contained 49 rice plants. Urea (200 kg/ha) and potassium chloride (60 kg/ha) were added to the soil following university recommendations (Hardke, 2018). All paddies were kept flooded to aid in transplantation until ~ 2 weeks after transplant when irrigation management treatments began.

Irrigation management treatments were designed to provide a range of soil redox conditions. Irrigation management included 4 different versions of AWD, a flooded control (“Flood”), and a nonflooded control (“Nonflooded”). For AWD treatments, the water table was allowed to drain to either 15 or 30 cm below ground surface prior to reflooding. The frequency of AWD dry downs was also varied, with paddies maintained under flooded conditions for 5 days prior to allowing the next dry down (i.e., low frequency AWD) or paddies allowed to immediately begin the next dry down after reflooding (i.e. high frequency AWD). The AWD treatments were abbreviated according to their depth of drainage and frequency of dry downs (e.g., AWD15HF). The Nonflooded treatment was watered, but not flooded, once the water table reached 30 cm below the ground surface. The water level in each paddy was measured using a nominally 5 cm diameter PVC pipe well. The pipe had 6 mm holes drilled every 2 cm of depth on 4 sides of the pipe. For making irrigation decisions, the water level was measured manually each day using a measuring tape. Irrigation decisions were made independently for each paddy after water level measurement. Each well also contained a water level logger that recorded the water level every 15 min (Hobo U20L-04, Onset Corp. Bourne, Massachusetts, USA). Irrigation management treatments were maintained until the paddies were drained for harvest, which was 100 and 105 days after transplant for years 1 and 2, respectively. High frequency AWD paddies had 9–13 dry downs each year while low frequency AWD paddies had 5–8 dry downs each year. Plants were harvested 111 and 112 days after transplant for years 1 and 2, respectively.

### Porewater and greenhouse gas sampling and analysis

Porewater was sampled weekly during the experiment using rhizon samplers. The rhizons (1910PL, Soil Moisture Corp. Golleta, California, USA) were installed 15 cm below the ground surface each week for sampling porewater. Porewater was collected either in a locking syringe or a degassed, anoxically sealed 20 mL vial. Porewater was immediately analyzed for pH and redox potential using calibrated probes (redox potentials reported relative to the standard hydrogen electrode). An aliquot of sample was also immediately pipetted for Fe(II) analysis by the ferrozine method (Stookey, 1970). The remaining porewater was acidified using trace metal grade nitric acid to 2% acid and analyzed for dissolved organic carbon (vario TOC cube) (Limmer & Seyfferth, 2021; Limmer et al., 2023), total elemental analysis (Thermo iCap ICP-MS), and arsenic speciation (IC-ICP-MS) (Jackson, 2015).

Soil redox potential was additionally measured continuously every 15 min at two depths using platinum (Pt) electrodes. The Pt electrodes consisted of a single probe inserted into the soil with Pt wires (Paleo Terra, Amsterdam, The Netherlands). The Pt wires were located 10 and 15 cm below the ground surface. In each paddy, a gel-filled KCl/AgCl reference electrode (Beckman Coulter A57193) was placed in a saturated KCl/agar salt bridge constructed from a 30 cm long PVC pipe. To make the agar solution, 30 g of agar was hydrated in 1 L of water, 350 g of KCl was added and the solution was brought to 90 °C. The solution was poured into the PVC pipe while hot and allowed to cool slightly prior to inserting the reference electrode. The Pt electrodes and reference electrodes were connected to differential inputs on a CR1000 data logger (Campbell Scientific, Logan, UT, USA), and redox potentials were recorded relative to the standard hydrogen electrode.

Measurements of CH_4_ fluxes occurred weekly throughout the growing season using the closed chamber technique. The chamber consisted of a cube (1.5 × 1.5 × 1.5 m) covered by translucent, woven polyethylene. The chamber provided an airtight seal of the entire paddy (soil and all 49 plants) and remained on each paddy for 5 min. The air inside the chamber was mixed by two 12 V DC fans and directed into a GasMet DX4040 that measured CH_4_ concentrations every 5 s. The linear portion of the CH_4_ concentration versus time curve was used to calculate the flux from the paddy surface area to chamber volume ratio after correcting for air temperature using the ideal gas law. The identification of the linear portion of the flux curve and unit conversions was performed in MATLAB (Limmer & Seyfferth, 2021; Limmer et al., 2018).

### Plant sampling and analysis

Plants were harvested at grain maturity and separated into different parts for chemical analysis. Rough rice was removed from the panicles and air-dried. The rough rice was dehusked and polished using a laboratory dehusker and polisher, respectively. Flag leaves and the 2 uppermost nodes were separated from the straw. Iron plaque was removed from the rice roots using a cold dithionite-citrate-bicarbonate extraction (Taylor & Crowder, 1983). All plant parts were finely ground prior to acid digestion using microwave digestion (Seyfferth et al., 2016). Briefly, ~ 0.2 g of plant material was combined with 7 mL of trace metal grade nitric acid in a microwave digestion vessel and held at 200 °C for 10 min (Mars 6, CEM Corp.). After cooling, the digest was diluted, centrifuged, and the acid fraction was transferred to a 50 mL centrifuge tube. The remaining Si-rich precipitate was washed, centrifuged, and decanted three times before being dissolved with 2 M NaOH. This solution was analyzed for Si using the molybdate blue method (Kraska & Breitenbeck, 2010). The acid fraction was diluted to 2% HNO_3_ and analyzed by ICP-MS (iCAP TQ, Thermo Fisher Scientific). The polished rice grain was analyzed for As speciation using a 2% HNO_3_ extraction (Maher et al., 2013) followed by IC-ICP-MS (iCAP TQ) (Jackson, 2015). Accuracy of plant elemental analysis was verified by analysis of certified reference materials: NIST 1568b (rice flour), NIST 1570a (spinach leaves), and WEPAL IPE 188 (oil palm). Arsenic speciation analysis was verified using NIST 1568b. Recoveries were acceptable for elements of interest (Table S2).

### Statistical analyses

The experimental design was a randomized complete block design, with a blocking factor for the age of the paddies. When the irrigation management effect was significant, mean comparisons were made using Tukey’s adjustment. Analysis was performed using SAS 9.4 and PROC GLIMMIX. Residuals were checked for normality and homoscedasticity. Porewater and water level data were averaged across the reproductive period (> 40 days after transplanting until draining) for statistical analysis.

## Results

### Water level and rice yield

**Fig. 1:**
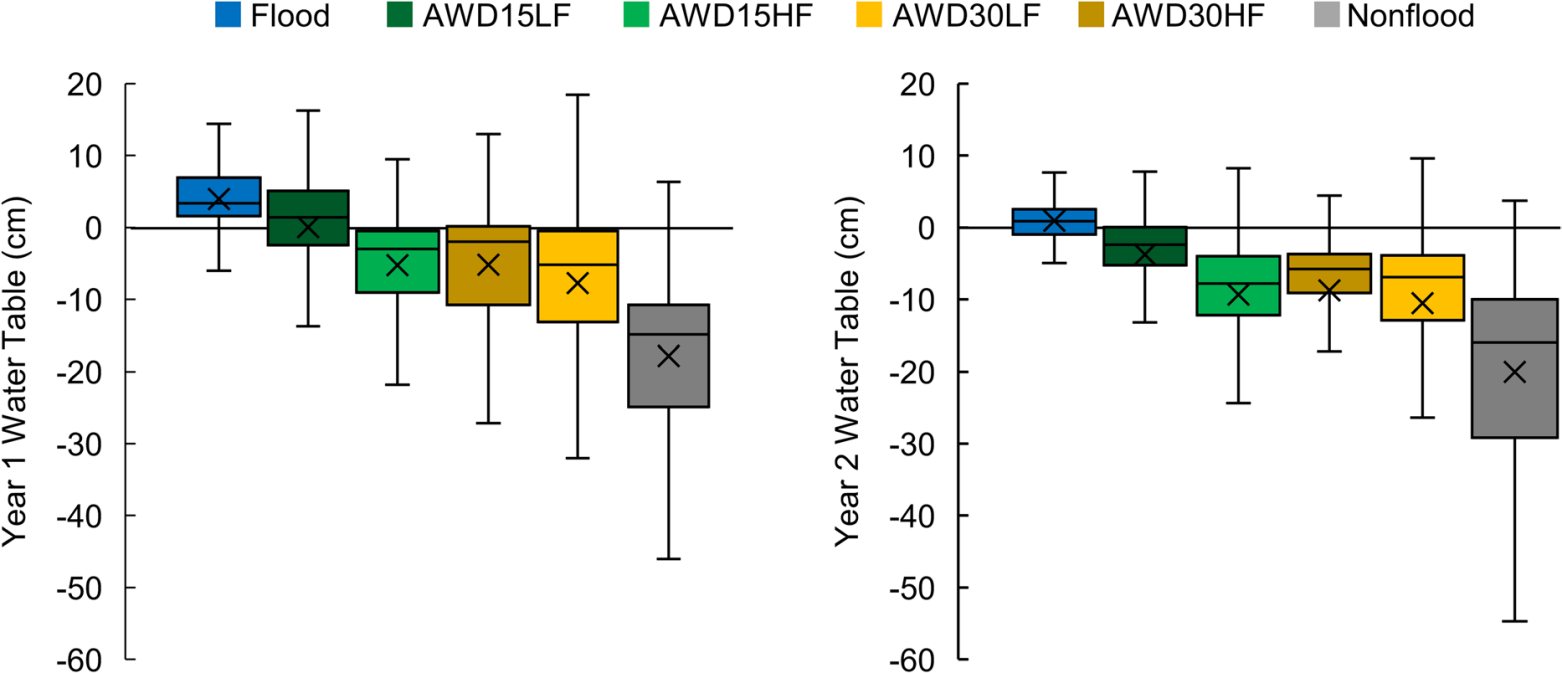
The effect of irrigation management treatments on the water table throughout the reproductive period (> 40 days after transplanting) and prior to harvest dry down. Boxes extend to the 25th and 75th percentiles, while error bars show the range of the data. The mean is denoted by an ‘x’ and the median is denoted by a line. Raw data are shown in Figure S1

The irrigation management treatments exposed the rice to a range of water levels. The flooded and nonflooded treatments bracketed the AWD treatments, with water levels under nonflooded conditions averaging ~ 20 cm below the ground surface (Fig. 1). Mean and median water levels generally decreased with increasing drainage frequency (“HF” < ”LF”) and increasing drainage depth (“30″ < ”15″). Irrigation management treatments were maintained throughout the majority of the growing season, resulting in consistent water level changes (Figure S1).

Irrigation management did not significantly affect straw biomass or rough rice yield in either year (Figure S2, Table S3). Straw tended to decrease under AWD30 and nonflooded treatments, but this effect was insignificant (*p* = 0.14 and *p* = 0.66 in years 1 and 2, respectively). Rough rice averaged 7250 kg/ha in year 1 and 6770 kg/ha in year 2, with yields decreasing with decreased flooding (*p* = 0.29 and *p* = 0.22 in years 1 and 2, respectively).

### Porewater and soil chemistry

**Fig. 2:**
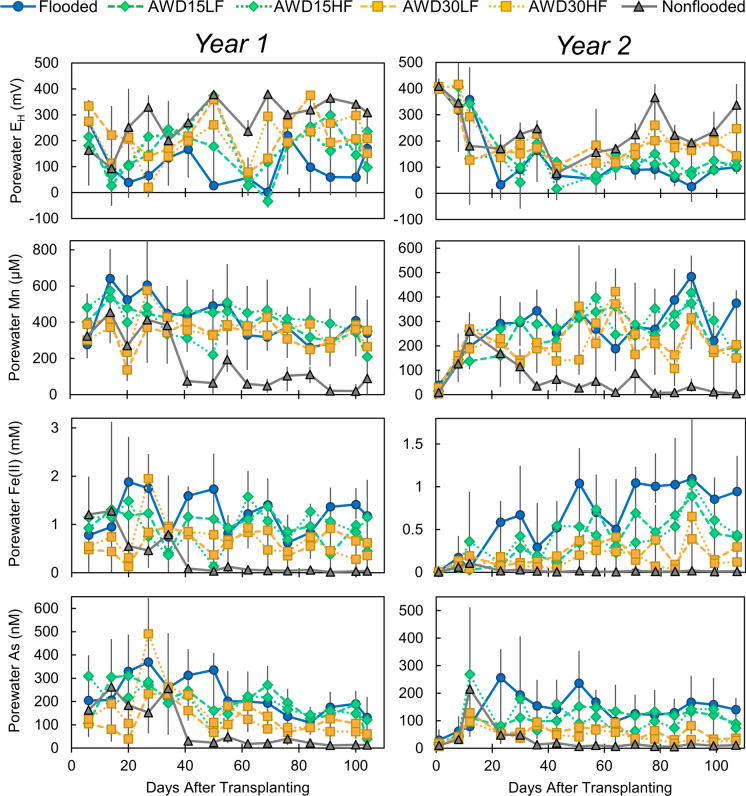
The effect of irrigation management on weekly porewater measurements of redox, Mn, Fe(II), and As. Error bars show the range of the data (n = 3). Average values and statistical analyses are given in Table S4

Irrigation management significantly affected the porewater concentrations of redox-sensitive elements (Fig. 2 and Fig. S3). In both years, flooded conditions resulted in the lowest porewater redox (E_H_), with average values during reproduction of 71 and 65 mV in year 1 and 2, respectively (Table S4). Nonflooded conditions resulted in the highest average reproductive E_H_ of 242 and 204 mV in year 1 and 2, respectively. In both years, AWD15 treatments had redox values not significantly different than flooded conditions, while AWD30 treatments were significantly different than both flooded and nonflooded treatments. Soil redox covered a wider range of values than porewater redox (Fig. S4). For the nonflooded treatment, soil E_H_ generally remained near 600 and temporarily dropped following rain/irrigation events, while the flooded treatment E_H_ generally remained < −200 mV. AWD treatments showed much more variability, with AWD30 treatments having higher E_H_ than AWD15 treatments. Except for nonflooded treatments, porewater Mn remained elevated for all treatments in both years. Porewater Fe(II) was strongly affected by irrigation management, with nonflooded conditions maintaining 95–99% lower Fe(II) concentrations compared to flooded conditions. No difference in average porewater Fe(II) was observed between LF and HF treatments, but AWD30 treatments resulted in lower concentrations of porewater Fe(II) than AWD15 treatments. Porewater As showed similar trends to porewater Fe(II), and average porewater As was well correlated with average porewater Fe(II) (r = 0.98 Figure S5). A vast majority of this As was inorganic As (As_i_), and more flooded conditions increased As_i_. In both years, < 10% of total As was organic As (As_o_). Organic arsenic included similar levels of TMAO, DMA, and MMA, and concentrations of As_o_ similarly increased under more flooded conditions but this effect was marginally significant (Table S4). Nonflooded conditions generally also led to significantly lower pH, Ca, and Mg and significantly higher S (Figure S3 and Table S4) compared to other irrigation managements. Porewater Cd, Zn, and Cu remained below the detection limit (10 nM, 1 μM, and 50 μM, respectively) for > 95% of the measurements.

### Methane emissions

**Fig. 3:**
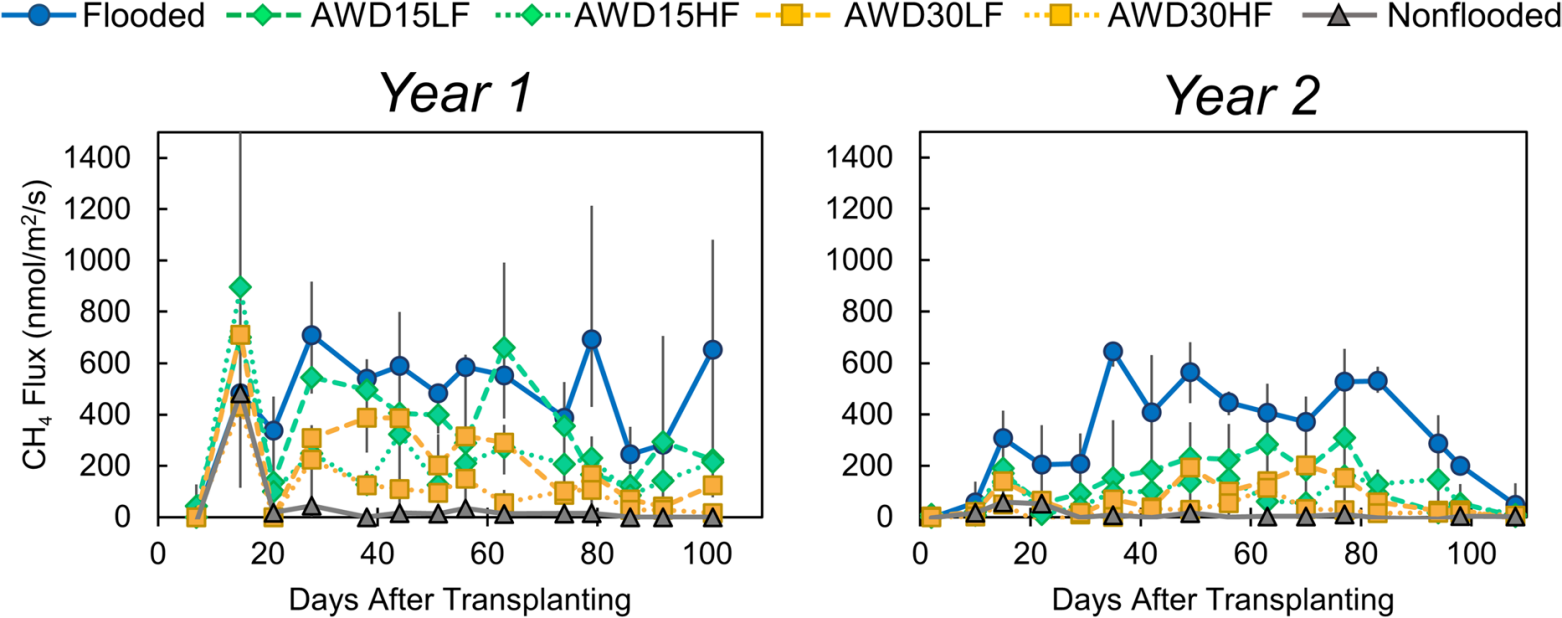
The effect of irrigation management on paddy methane emissions measured weekly through the growing season. Error bars show the range of the data (n = 3). Average values and statistical analyses are given in Table S4

Irrigation management strongly affected cumulative CH_4_ emissions (Fig. 3). Methane emissions were highest for the flooded treatment, reaching 3900 mmol/m^2^ in year 1 and 3100 mmol/m^2^ in year 2 (Table S4). In year 1, AWD15LF decreased methane emissions by 21%, while all other treatments decreased CH_4_ emissions by 51–90%. In year 2, alternative irrigation managements decreased methane emissions more strongly relative to flooded conditions (60–97%). The average water level was significantly correlated to cumulative CH_4_ emissions across both years, except for nonflooded conditions (r = 0.90, Fig. S6. More aerobic irrigation managements also tended to decrease porewater DOC, although this effect was only significant in year 2 (Table S4).

### Plant As

**Fig. 4:**
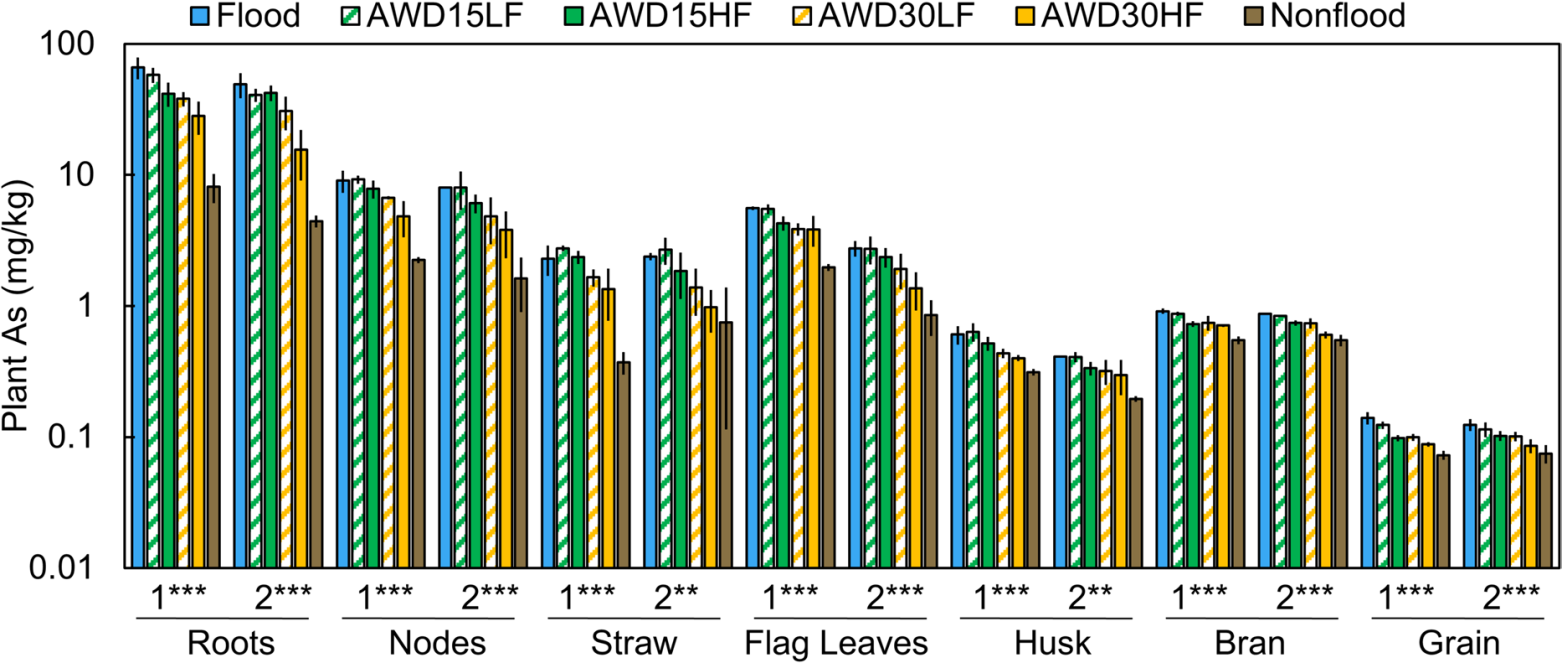
Effect of irrigation management on total As concentrations in various plant parts separated by year 1 and year 2 of the study. The number of asterisks for each year denote the significance of irrigation management on plant As for each part (*p < 0.05, **p < 0.01, ***p < 0.001). Error bars show the standard deviation (n = 3). Note data are shown on a log scale. Average values and mean comparisons are given in Table S5

Plant arsenic concentrations were strongly affected by irrigation management in all plant parts measured (*p* < 0.01, Fig. 4). Concentrations of As in all plant parts tended to decrease with increasingly aerobic irrigation management. However, AWD15LF and Flooded treatments never significantly differed for any plant part in either year (Table S5). Comparisons of dry down frequency within a dry down severity (e.g., AWD15LF vs. AWD15HF) rarely showed significant differences in As concentrations.

**Fig. 5:**
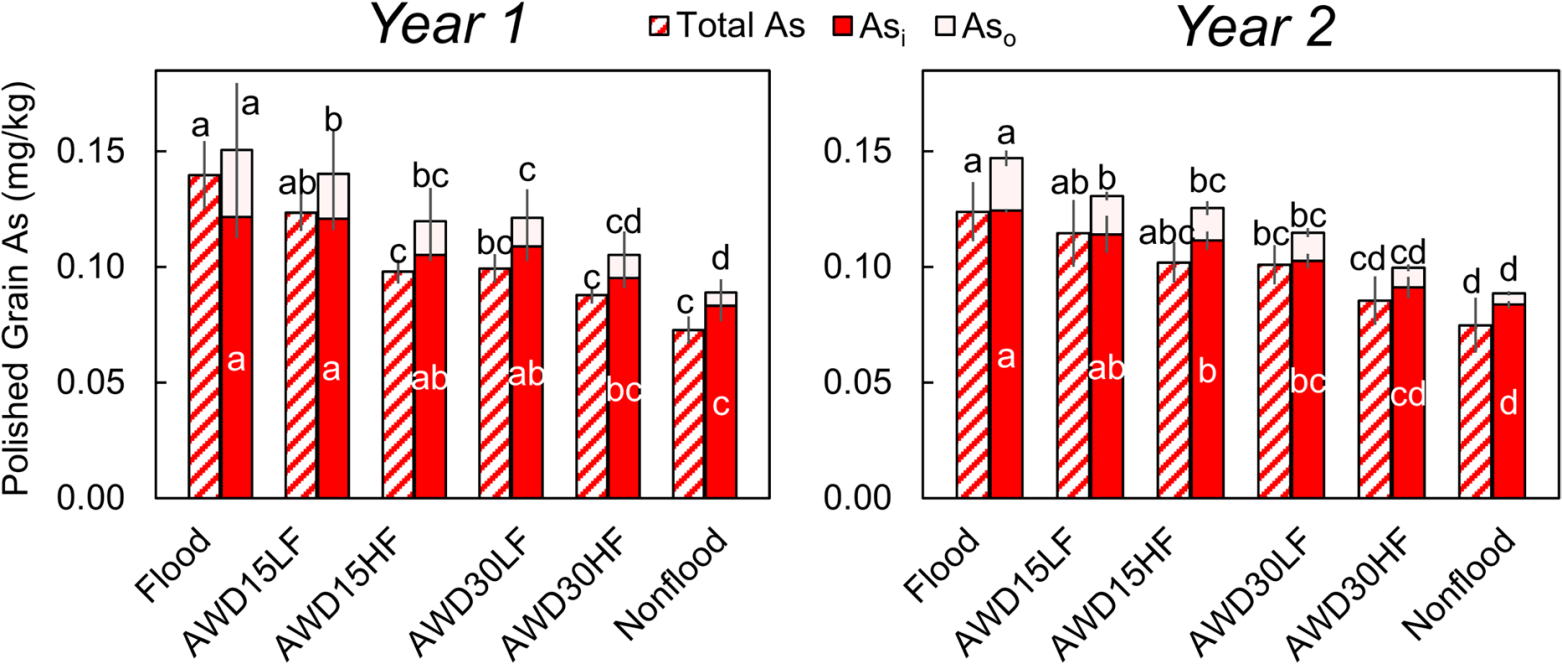
Effect of irrigation management on rice grain As speciation. Statistics were performed separately for each As species and each year. Bars with different letters are statistically different at α = 0.05. Error bars show the standard deviation (n = 3). Average values are given in Table S6

**Fig. 6:**
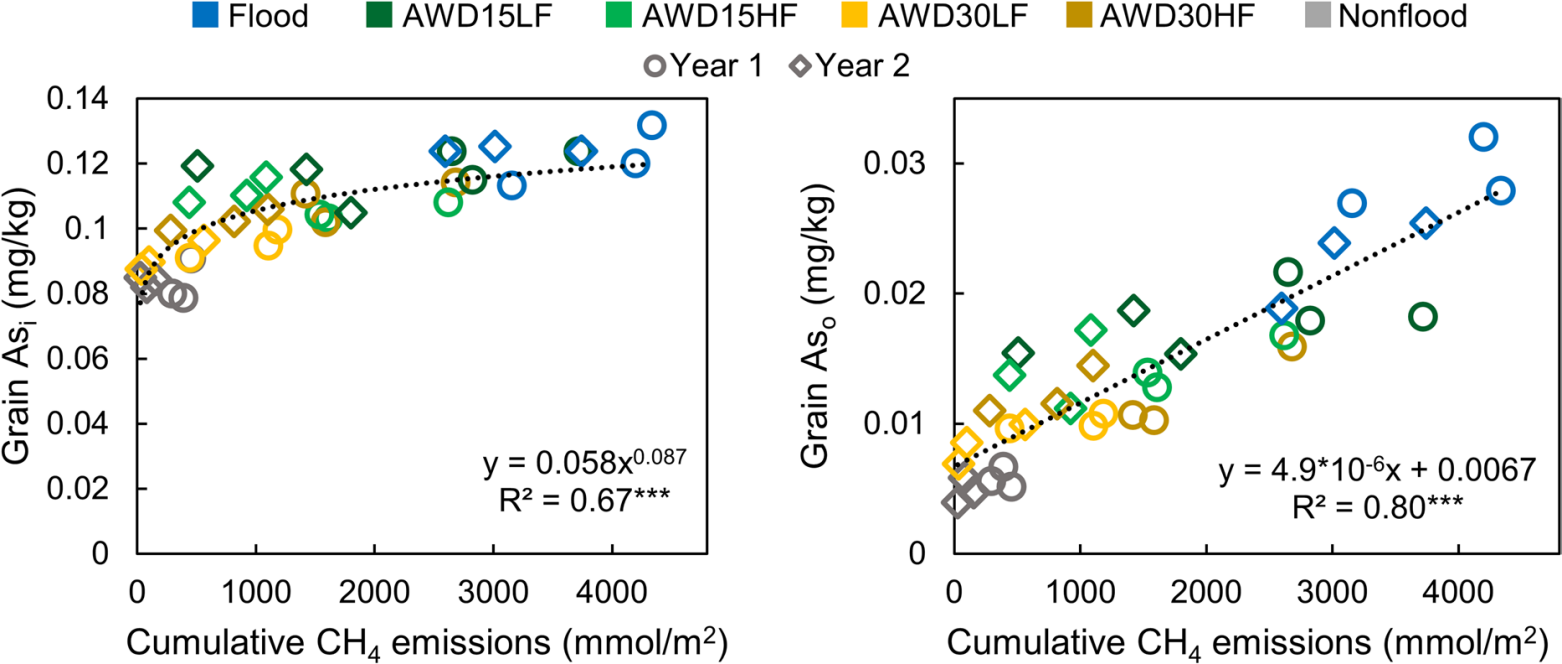
Correlation between cumulative methane emissions and grain As speciation for both years of the experiment

Polished rice grain concentration and speciation of As were strongly affected by irrigation management in both years (Fig. 5). Total As and As speciation generally decreased as irrigation management became more aerobic; however, AWD15LF did not significantly differ from flooded in total As or inorganic As (Table S6). The effect of irrigation management on As_o_ was stronger, with all irrigation managements decreasing As_o_ 27–80% relative to the flooded treatment (*p* < 0.0001). In contrast, aerobic irrigation managements only decreased As_i_ by at most 33% relative to the flooded treatment (*p* < 0.0001). In these samples, As_o_ was predominantly DMA with traces of MMA and TMAO. However, the As extraction method could not preserve dimethylmonothioarsenate (DMMTA), which would have been converted to DMA during the extraction (Colina Blanco et al., 2021). The strong effect of irrigation management on grain As_o_ is also evident in the strong linear relationship with cumulative CH_4_ emissions (r = 0.90, Fig. 6). While grain As_i_ was also positively correlated with cumulative CH_4_ emissions, the relationship was nonlinear, with aerobic irrigation management decreasing CH_4_ emissions faster than grain As_i_ (Fig. 6). Grain As also showed strong correlations with porewater Fe (r = 0.72) and porewater E_H_ (r = -0.72, Fig S7).

### Plant Cd

**Fig. 7:**
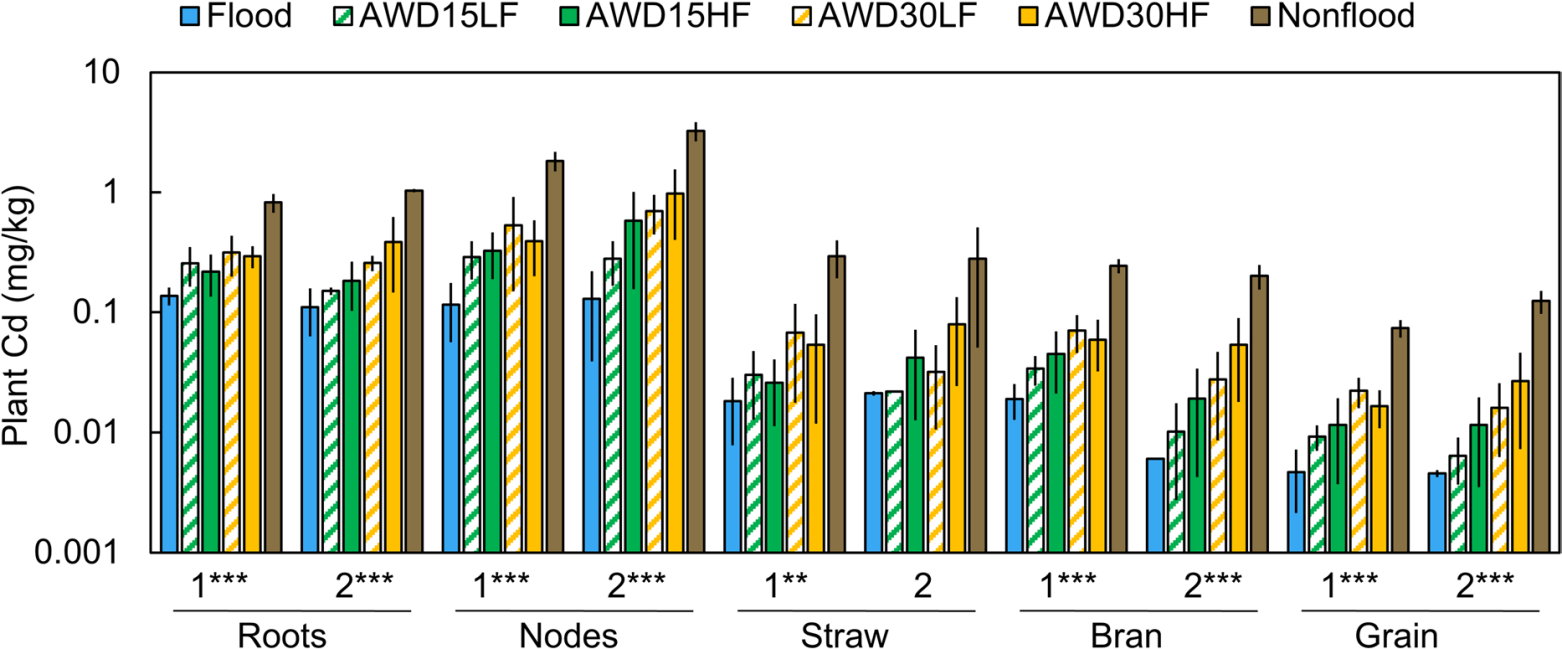
Effect of irrigation management on Cd concentrations in various plant parts separated by year 1 and year 2. The number of asterisks for each year denote the significance of irrigation management on plant Cd for each part (*p < 0.05, **p < 0.01, ***p < 0.001). Error bars show the standard deviation (n = 3). Note data are shown on a log scale. Average values and mean comparisons are given in Table S7

**Fig. 8:**
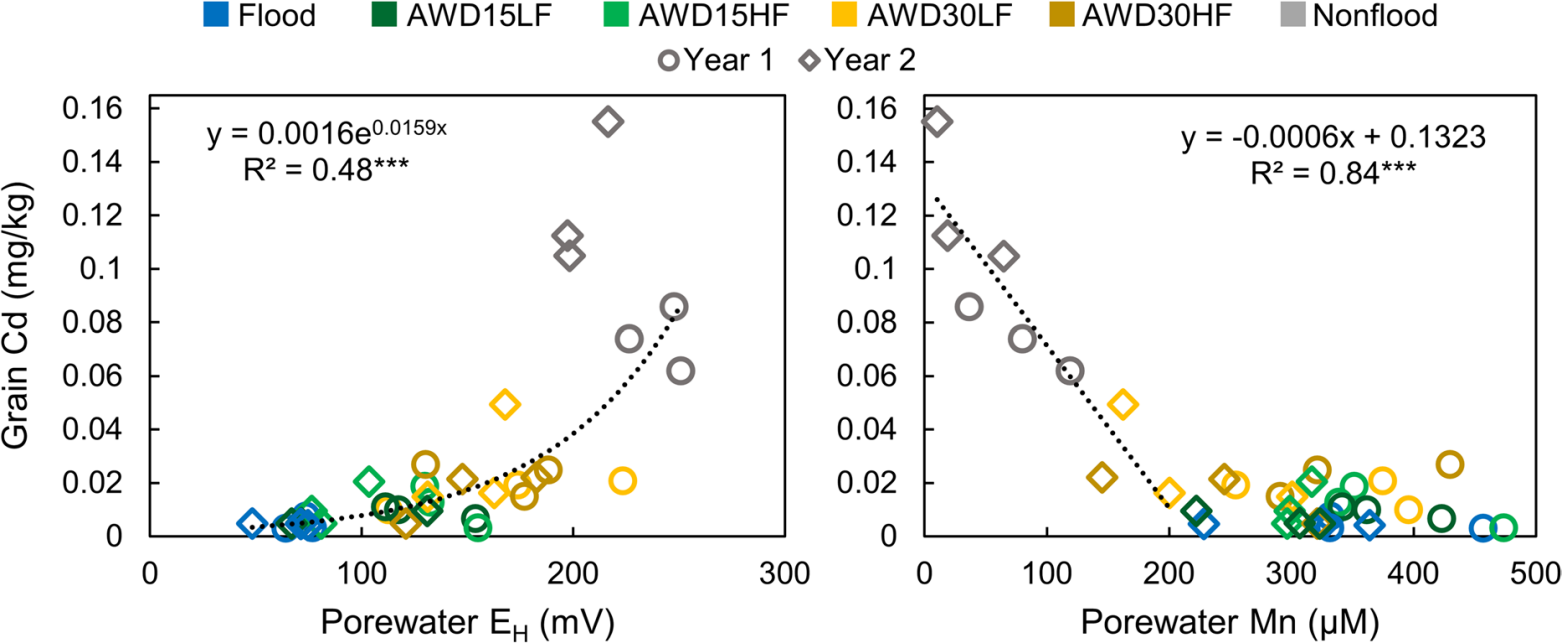
Correlations between grain Cd and porewater redox and porewater Mn across all irrigation managements and both years. Porewater values were averaged across the reproductive period

Plant Cd concentrations were strongly and consistently affected by irrigation management (Fig. 7). In all plant parts, the flooded treatment contained the lowest plant Cd concentrations while nonflooded contained the highest plant Cd concentrations. Cadmium concentrations decreased from the roots toward the grain and concentrations were less than the detection limit in the flag leaves and husk. Concentrations of Cd in the polished grain increased by ~ 1 order of magnitude from flooded to nonflooded conditions. However, porewater and soil E_H_ were not particularly strong predictors of Cd in polished grain because grain Cd increased faster than E_H_ under more aerobic conditions (Fig. 8 and Fig. S8). Instead, porewater Mn was well correlated with grain Cd (R^2^ = 0.84, Fig. 8), but only in paddies where average porewater Mn remained below 200 μM did grain Cd increase inversely to porewater Mn.

### Plant nutrients

Irrigation management had variable effects on the concentrations of other plant nutrients. Plant P tended to decrease under more aerobic conditions, particularly in the roots, nodes, and straw (Fig. S9 and Table S8). Plant K showed declining concentrations with more aerobic treatments in the nodes, flag leaves, and husk (Fig. S11 and Table S9). For Si, which was readily accumulated in straw and husk, irrigation management never significantly affected Si concentrations in any plant part (Fig. S11 and Table S10). Plant Cu behaved similarly to Cd, with increasing concentrations in most plant parts under increasingly aerobic irrigation managements (Fig. S12 and Table S11). Despite the strong effect of irrigation management on porewater Fe, plant Fe remained relatively stable, with concentrations of Fe only elevated under more flooded conditions in the roots and nodes (Table S12 and Fig. S13). Plant Mg was rarely significantly affected by irrigation management, although the trend was for decreasing plant Mg concentrations under more aerobic irrigation managements (Fig. S14 and Table S13). In contrast, increasingly aerobic irrigation managements increased plant Mn in most aboveground parts (Fig. S15 and Table S14). This effect was most pronounced in the flag leaves, which, under nonflooded conditions, contained more than double the Mn under flooded conditions. Plant Zn was generally unaffected by irrigation management (Fig. S16 and Table S15).

## Discussion

### Irrigation management and grain As

Irrigation management strongly affected rice accumulation of As, although effects on As species varied. Arsenic in all measured plant parts significantly increased under more flooded conditions in both years (Fig. 4). The more flooded treatments exhibited Fe(III)-reducing conditions, as evidenced by the low porewater redox potential, soil redox potential, and high porewater Fe(II) concentrations (Fig. 2 and Fig. S4). Such conditions resulted in higher porewater As, and this As was well correlated with porewater Fe (Fig. S5) due to the Fe(III)-reducing conditions (Bose & Sharma, 2002). While grain total As significantly decreased with drier irrigation managements, drier irrigation managements more strongly decreased grain organic As than grain inorganic As (Fig. 5). This is in agreement with a recent review that found that when paddies are dried down, grain total As decreases more rapidly than inorganic As (Carrijo et al., 2022). Thus, stronger aerobic conditions would have been needed to decrease grain inorganic As. Additionally, arsenic in all plant parts did not significantly differ between flooded and AWD15LF (Table S5), the latter resembling “safe AWD” which is the form of AWD most frequently recommended to prevent the yield losses that can occur under more severe dry downs (Bouman, Lampayan, et al., 2007). Thus, it is unlikely that the recommended dry down intensity will result in appreciable declines in grain inorganic As.

The speciation of As in rice grain showed different correlations to CH_4_ emissions, providing insight into the mechanisms underlying As methylation and their dependence on irrigation management. Grain inorganic As showed a nonlinear relationship with cumulative CH_4_ emissions, with grain inorganic As remaining relatively constant when cumulative CH_4_ emissions exceeded 1000 mmol/m^2^ and only decreasing more sharply when CH_4_ emissions decreased further (Fig. 6). In contrast, grain organic As was linearly and positively correlated with cumulative CH_4_ emissions and the intercept of the model was an order of magnitude lower than modeled for inorganic As (Fig. 6). Methylation of inorganic As occurs under low redox conditions (Dykes et al., 2021), with some sulfate-reducing bacteria (Viacava et al., 2022; Wang et al., 2015) and methanogens (Viacava et al., 2020; Wang et al., 2014) able to methylate inorganic As. However, the amount of As_o_ is dependent on the relative rates of microbial methylation and demethylation. Methanogens, particularly methylotrophic methanogens are responsible for demethylating As (Chen et al., 2019, 2023; Zhang & Reid, 2022). The positive relationship observed here between CH_4_ emissions and grain As_o_ suggests that in this soil as soil redox potentials decreased, both the activity of methanogens and As methylators increased, and also that the rate of As methylation outpaced the rate of demethylation as irrigation managements became more flooded.

### Irrigation management and grain Cd

Increasingly aerobic forms of irrigation management led to increased plant concentrations of Cd. In both years, cadmium increased in all plant parts as irrigation managements became more aerobic, with nonflooded conditions leading to the highest Cd (Fig. 7). Cadmium is well known to be more mobile under drier conditions in acidic soils as the soil oxidizes, and some have attributed this effect to the decrease in porewater pH resulting from the production of protons during Mn(II) and Fe(II) oxidation (Wang et al., 2019). However, here we did not consistently observe a substantially lower porewater pH under nonflooded conditions (Figs. S3 and S17). Oxidation of CdS is another possible cause of increased Cd availability, although the oxidation of CdS appears to be relatively slow (de Livera et al., 2011; Wang et al., 2019; Yang et al., 2021). Additionally, we did not observe a particularly strong relationship between porewater redox potential and grain Cd, suggesting that redox potential may not be the best predictor of Cd availability (Fig. 8).

In addition to the above factors, Cd accumulation by rice also interacts with Mn in several ways. First, Mn(II) oxidizes when paddies are drained, and these Mn oxides are strong oxidants capable of oxidizing many of the reduced substances in the soil, such as CdS. Second, Mn oxides can adsorb Cd, which would be expected to decrease the uptake of Cd. A multiple linear regression model found that the best prediction of grain Cd required both soil pH and soil amorphous Mn and these were negatively correlated with grain Cd (Wang et al., 2020). Manganese also plays a direct role in the uptake of Cd because Cd enters the rice root through the Mn transporter OsNramp5 (Ishimaru et al., 2012; Sasaki et al., 2012). Manganese can compete with Cd for uptake (Q. Huang et al., 2017), and several studies have found that application of Mn fertilizers and Mn-rich soil amendments decrease grain Cd (Fang et al., 2021; Liang et al., 2022). Such Mn fertilizers have been reported to increase the porewater Mn and decrease plant Cd (G. Huang et al., 2021a, b, c). Here, we instead observed a negative correlation with porewater Mn and grain Cd (Fig. 8). This results from irrigation management controlling both Mn and Cd availability. As the soils dried, porewater Mn decreased due to the formation of Mn oxides while available Cd was likely also increasing. The strong correlation observed here may indicate similarities in the kinetics of Mn oxidation and Cd availability. The oxidation rate of Mn(II) in paddy porewater can vary. For example, in an Australian vertosol and sodosol, Mn(II) in the porewater varied with sampling depth, but trends were generally similar in the rooting depth of 7.5 cm. However, the porewater Mn(II) in the two different soils differed upon post-harvest drying, with the smectite clays in the vertosol shrinking and causing soil cracking, which quickly decreased porewater Mn(II) in the shallow porewater while the porewater Mn(II) took longer to decrease in the sodosol (Haque et al., 2015). Furthermore, while porewater Mn was well-correlated with grain Cd in this soil, it is unlikely to be correlated across different soils given the range of soil Mn, but porewater Mn(II) may still be a valuable predictor if normalized by a measure of soil reducible Mn.

### Tradeoffs between As and Cd

Finding the ideal level of irrigation management to simultaneously minimize grain As and Cd has proven difficult. Several authors have attempted to minimize both Cd and As by irrigation management, but generally observed a tradeoff between As and Cd with very few observations able to simultaneously minimize both As and Cd (Honma et al., 2016; Linam et al., 2022; Seyfferth et al., 2019; Yao et al., 2022). Both As and Cd are accumulated by rice during grain filling, but As accumulation begins earlier than Cd (Arao et al., 2009; Li et al., 2009), so extremely careful timing of irrigation management may be able to simultaneously minimize grain As and Cd. Alternatively, a recent meta-analysis suggested that flooding with severe dry downs appear to be required for the simultaneous minimization of grain Cd and As (Carrijo et al., 2022), although such dry downs may put rice at a risk for yield loss, particularly when performed during grain filling (Carrijo et al., 2017; Hardke, 2018).

While redox reactions broadly control rice accumulation of As and Cd, soil-specific factors are important considerations when determining an optimal approach to minimizing the risk posed by As and Cd. Because grain As is strongly related to Fe(III) reduction, the concentration of As associated with reducible Fe is an important screening factor. The rate of Fe(III) reduction in different soils can vary, thereby affecting the exposure of the rice plant to porewater As. For Cd, flooded conditions decrease the soil redox potential, which in acidic soils raises the soil pH, promotes sulfide formation, and increases soil organic matter concentrations, all of which decrease the availability of Cd (de Livera et al., 2011; Smolders & Mertens, 2013; Yang et al., 2021; Yuan et al., 2021). Thus, soil properties such as pH, available sulfur, and soil organic matter can affect Cd availability in addition to extractable levels of Cd in soils.

**Fig. 9:**
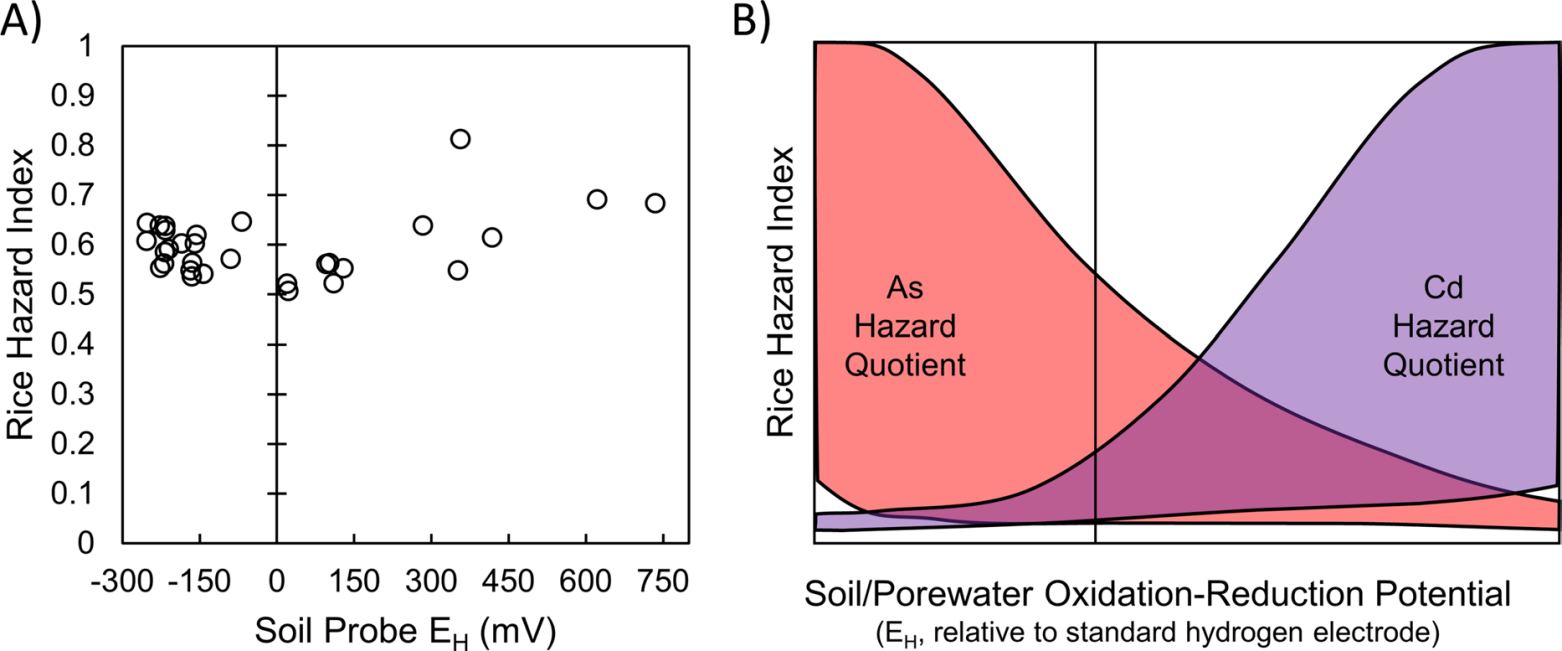
The effect of redox on the rice hazard index. **A** The data collected in this study show no notable trend with redox because the tradeoff between As and Cd was comparable in magnitude. **B** A conceptual diagram of how redox can affect the rice hazard index. Several soil factors affect the magnitude of the contribution to the hazard index from As and Cd, but a minimum may be possible at intermediate values of redox

Thus, soils may be at risk for rice accumulation of As, Cd, both, or neither. In plotting tradeoff curves in previous literature, the y-axis has either been in units of Cd or As (mg/kg) or scaled by the maximum value measured (Honma et al., 2016; Linam et al., 2022; Yao et al., 2022). We suggest that rather than plotting As and Cd separately, combining the grain data using a hazard index approach using the hazard quotients for inorganic As and Cd:

$$ HI = \frac{{\left[ {As} \right]}}{{0.2{\text{ mg}}/{\text{kg}}}} + \frac{{\left[ {Cd} \right]}}{{0.4 \;{\text{mg}}/{\text{kg}}}} $$

where HI is the hazard index, [As] is the concentration of inorganic As in the polished grain (mg/kg), [Cd] is the concentration of Cd in the polished grain (mg/kg), and 0.2 mg/kg and 0.4 mg/kg are the CODEX limits for the respective elements. In contrast to our hypothesis that we could achieve redox conditions that minimized both As and Cd, the effect of redox conditions had very minimal effect on the HI for the irrigation managements tested, and the HI remained below 1 in all cases (Fig. 9A). We hypothesized that water managements that were dominated by Mn reduction but without substantial Fe reduction could minimize both As and Cd. In our soil Mn reduction and Fe reduction substantially overlapped, so no such redox regime was achieved. This may be due to the relatively low concentrations of both soil As and Cd and/or the low reducible soil Mn/Fe in the present study. Conceptually, we still expect that in many cases there will be a tradeoff between As and Cd, but the wide range of soil properties gives a very wide range of potential hazard quotients for As and Cd (Fig. 9B). In some soils, the HI may be dominated by either As or Cd, and the mechanism may vary between soils, even those with similar HI values. Additionally, this HI approach only considers inorganic As, but the toxic dimethylmonothioarsenate (DMMTA) is present in rice and is thought to be more toxic than inorganic As (Colina Blanco et al., 2021; Planer-Friedrich et al., 2022). The addition of DMMTA to the HI will likely shift the minimum HI point as a function of redox conditions. Additional studies are needed to better predict the HI as a function of soil properties and irrigation management.

## Supplementary Information

Below is the link to the electronic supplementary material.

Supplementary file1 (DOCX 6422 kb)

Supplementary file2 (XLSX 8986 kb)
