# Supplementary material for: Controlling exposure to As and Cd from rice via irrigation management
Source: Environ Geochem Health. 2024 Jul 29;46(9):339. doi: 10.1007/s10653-024-02116-x (PMC11286649; doi:10.1007/s10653-024-02116-x)
Supplement: Supplementary file 2 — Supplementary file2 (XLSX 8986 kb) [file 10653_2024_2116_MOESM2_ESM.docx]

SI: Controlling exposure to As and Cd from rice via irrigation management

Matt A. Limmer and Angelia L. Seyfferth*

University of Delaware, Department of Plant and Soil Science, Newark, DE USA

# Supplemental Figures

Figure S1. Water table measured in rice paddies for both years S4

Figure S2. Effect of water management on straw biomass and rough rice yield S5

Figure S3. The effect of water management on weekly porewater measurements S6

Figure S4. Soil redox measured in rice paddies for both years S7

Figure S5. Correlation between average porewater Fe and As. S8

Figure S6. Correlation between cumulative methane emission and average water table for both years of the experiment S8

Figure S7. Correlations between grain As and porewater Fe and porewater redox across all water managements and both years.. S9

Figure S8. Trade off plots for grain As and Cd against porewater or soil redox. S9

Figure S9. Effect of water management on P concentrations in various plant parts S10

Figure S10. Effect of water management on K concentrations in various plant parts S10

Figure S11. Effect of water management on Si concentrations in various plant parts S11

Figure S12. Effect of water management on Cu concentrations in various plant parts S11

Figure S13. Effect of water management on Fe concentrations in various plant parts. S12

Figure S14. Effect of water management on Mg concentrations in various plant parts. S12

Figure S15. Effect of water management on Mn concentrations in various plant parts S13

Figure S16. Effect of water management on Zn concentrations in various plant parts S13

Figure S17. Correlations between grain Cd and porewater DOC and porewater pH across all water managements and both years. S14

# Supplemental Tables

Table S1. Soil elemental composition (mg/kg) as measured by total extraction (EPA 3051) and plant available nutrients (Mehlich-3) S15

Table S2. Analysis of certified reference materials.. S15

Table S3. Plant straw biomass and rough rice yield S16

Table S4. Average values for constituents measured during each year of the experiment S17

Table S5. Plant As concentrations S18

Table S6. Polished grain As species S19

Table S7. Plant Cd concentrations S19

Table S8. Plant P concentrations S20

Table S9. Plant K concentrations S21

Table S10. Plant Si concentrations S21

Table S11. Plant Cu concentrations S22

Table S12. Plant Fe concentrations S23

Table S13. Plant Mg concentrations S24

Table S14. Plant Mn concentrations S25

Table S15. Plant Zn concentrations S26

# Supplemental Figures


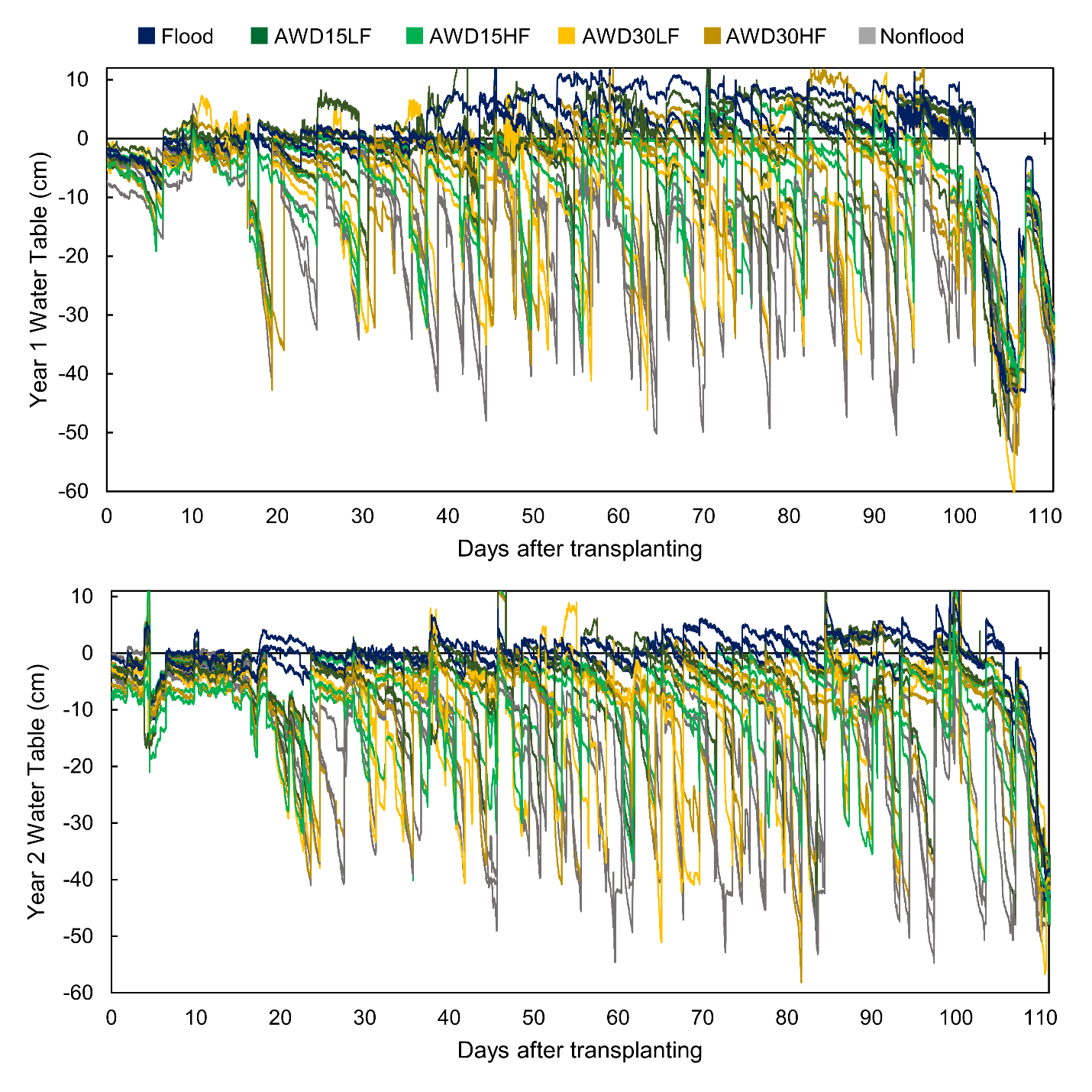


Figure S1. Water table measured in rice paddies for both years. All replicates are shown in both plots.


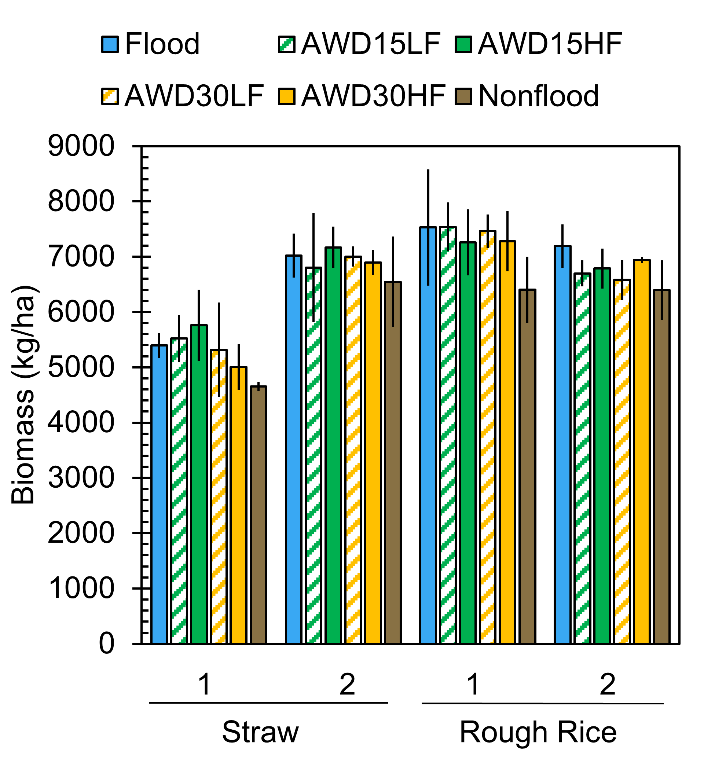


Figure S2. Effect of irrigation management on straw biomass and rough rice yield separated by year. The number of asterisks for each year denote the significance of irrigation management on plant Si for each (*p<0.05, **p<0.01, ***p<0.001). Error bars show the standard deviation (n=3).


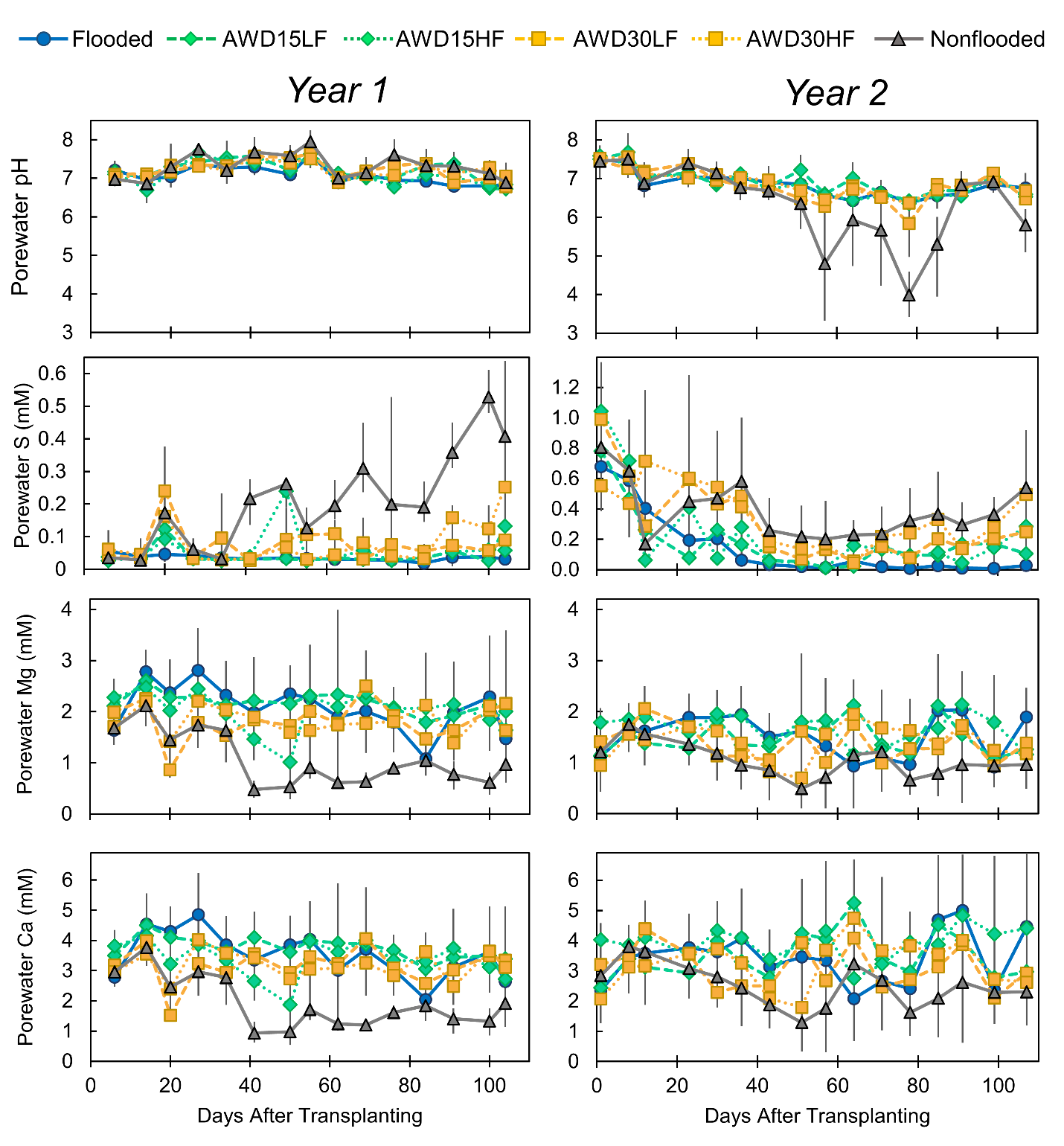


Figure S3. The effect of irrigation management on weekly porewater measurements of pH, S, Mg, and Ca. Error bars show the range of the data (n=3).


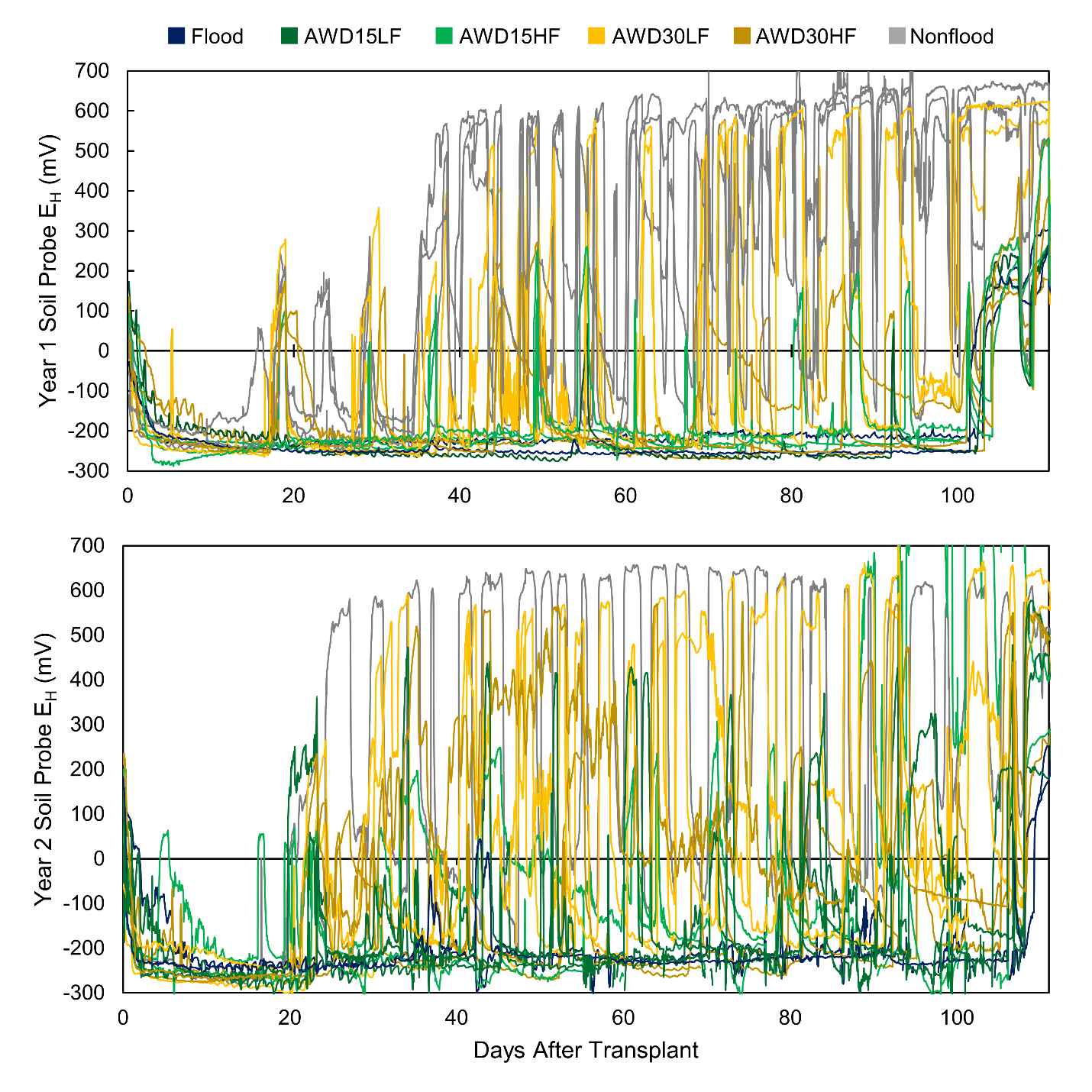


Figure S4. Soil redox measured in rice paddies for both years. All replicates are shown in both plots.


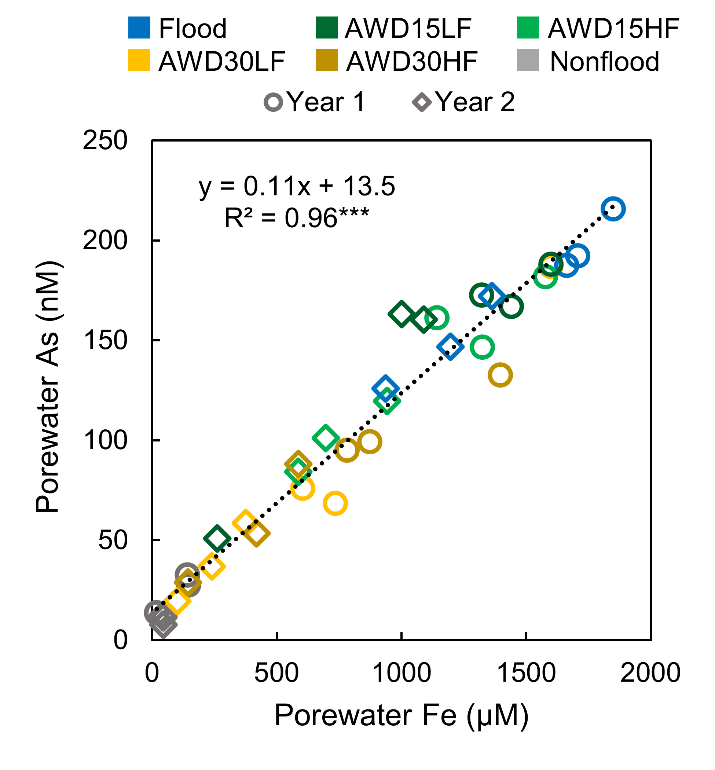


Figure S5. Correlation between average porewater Fe and As for both years of the experiment.


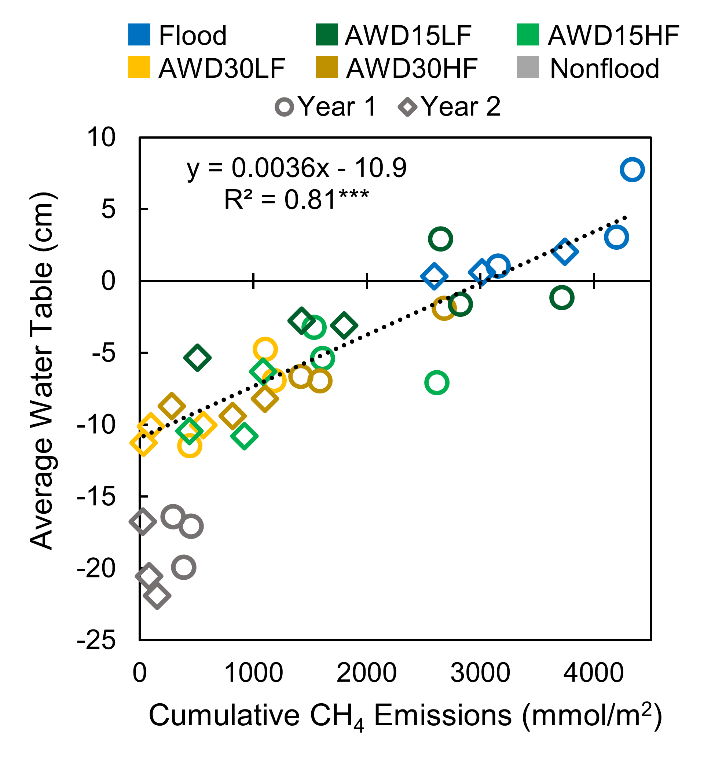


Figure S6. Correlation between cumulative methane emission and average water table for both years of the experiment. Regression fit excludes nonflooded treatment.


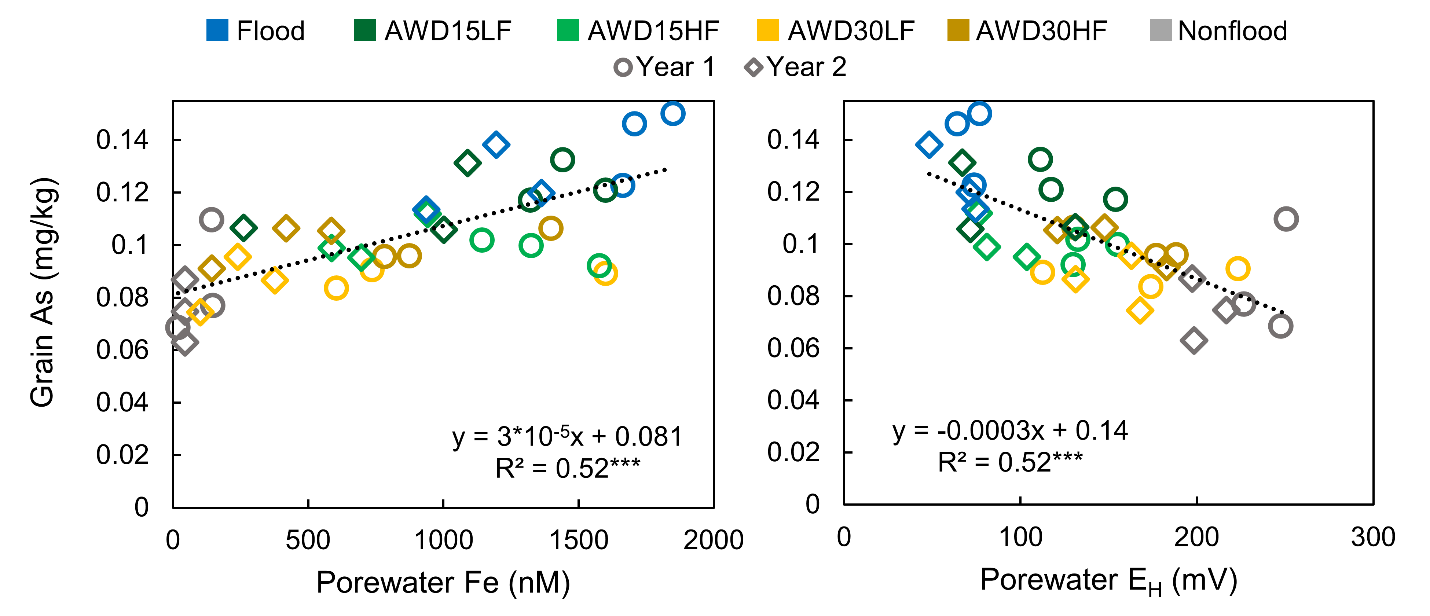


Figure S7. Correlations between grain As and porewater Fe and porewater redox across all irrigation managements and both years. Porewater values were averaged across the rice reproductive period.


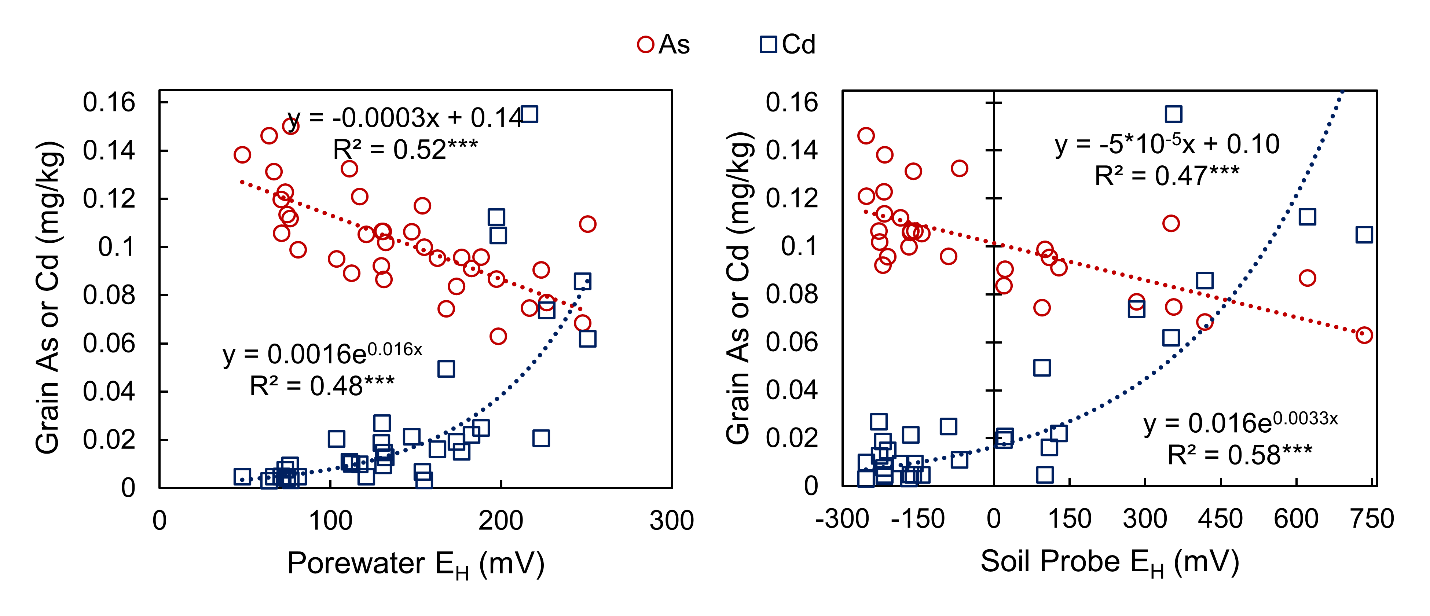


Figure S8. Trade off plots for grain As and Cd against porewater or soil redox.


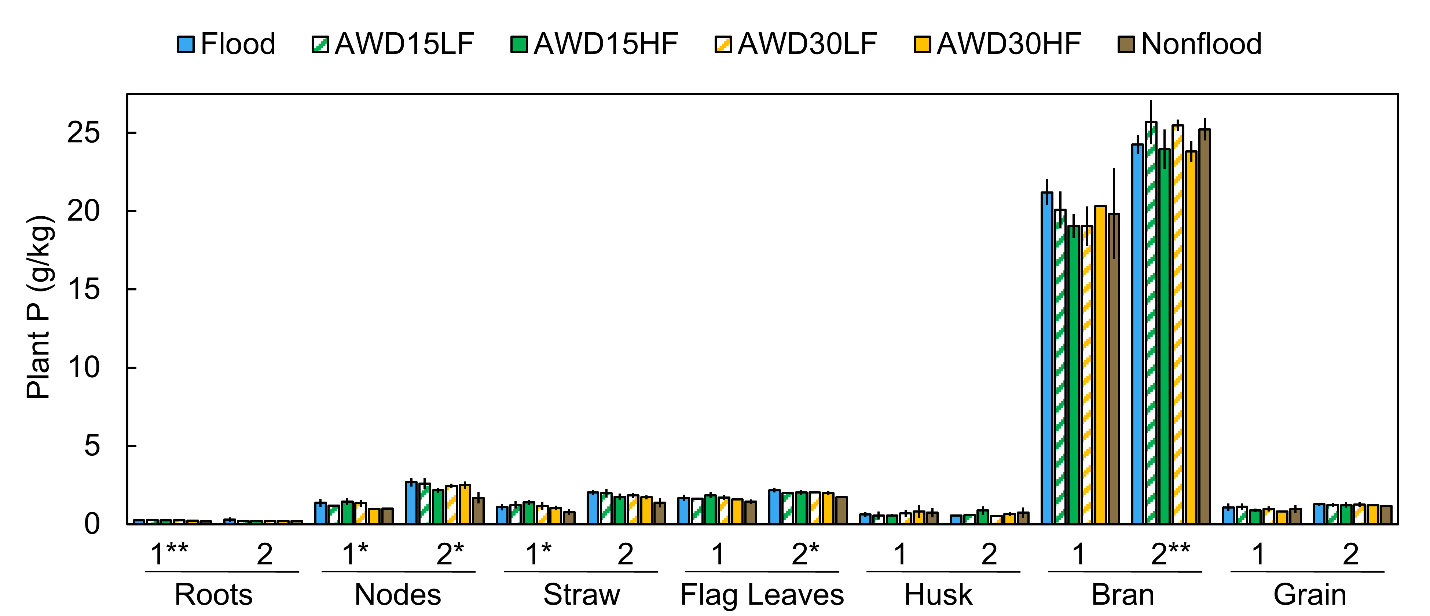


Figure S9. Effect of irrigation management on P concentrations in various plant parts. The number of asterisks for each year denote the significance of irrigation management on plant P for each part (*p<0.05, **p<0.01, ***p<0.001). Error bars show the standard deviation (n=3).


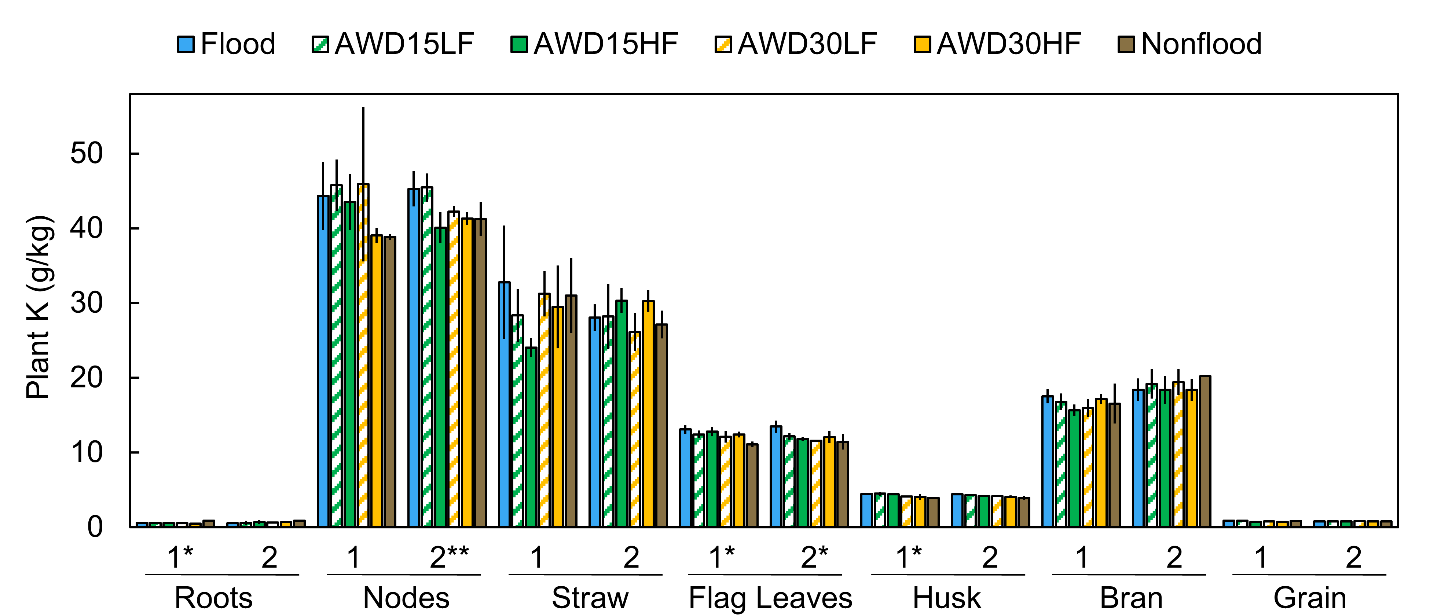


Figure S10. Effect of irrigation management on K concentrations in various plant parts. The number of asterisks for each year denote the significance of irrigation management on plant K for each part (*p<0.05, **p<0.01, ***p<0.001). Error bars show the standard deviation (n=3).


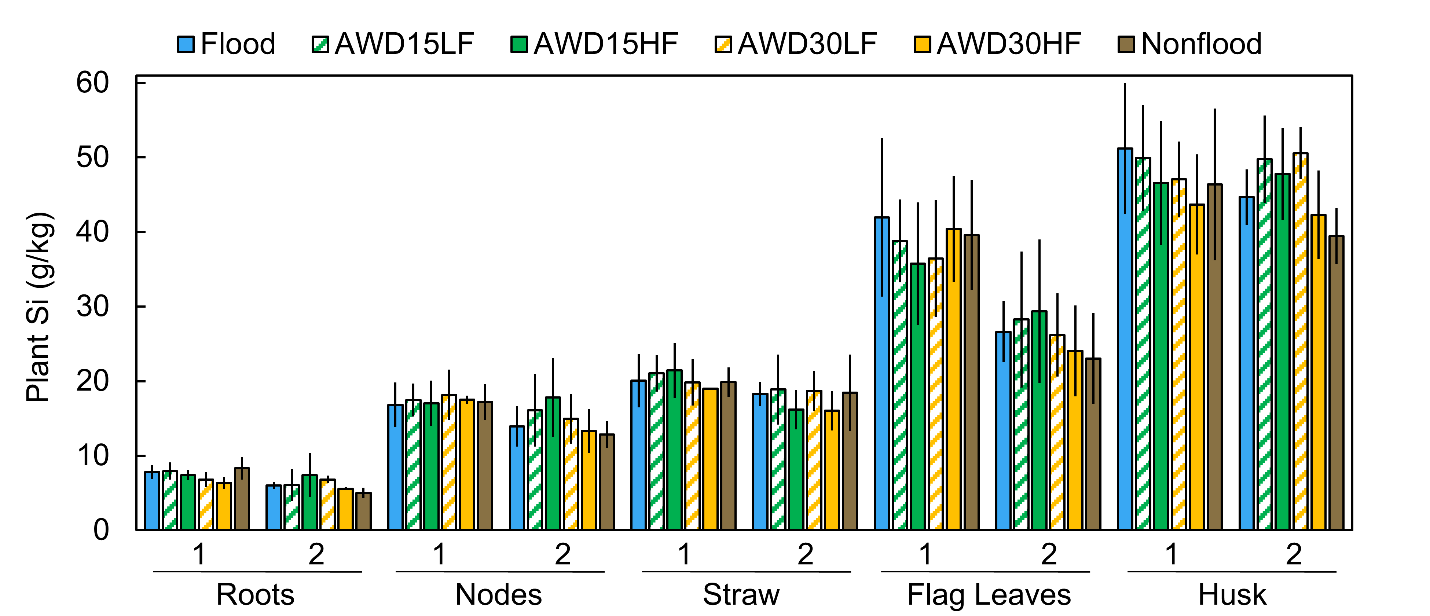


Figure S11. Effect of irrigation management on Si concentrations in various plant parts. The number of asterisks for each year denote the significance of irrigation management on plant Si for each part (*p<0.05, **p<0.01, ***p<0.001). Error bars show the standard deviation (n=3).


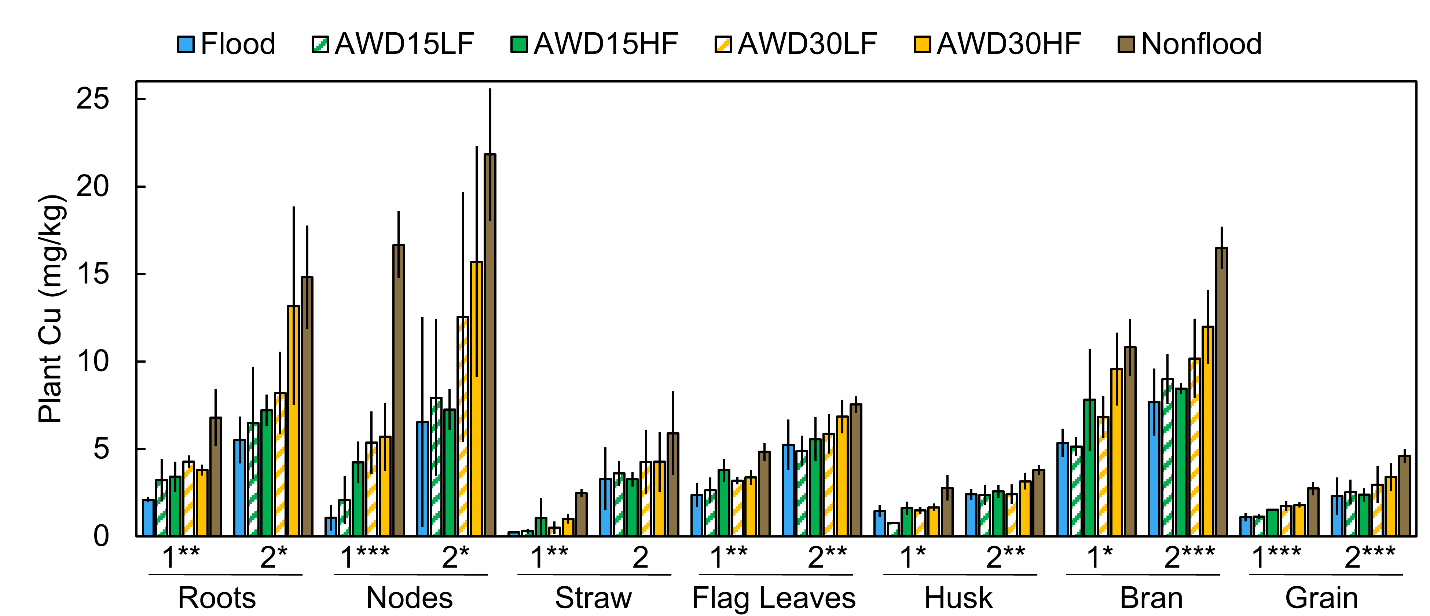


Figure S12. Effect of irrigation management on Cu concentrations in various plant parts. The number of asterisks for each year denote the significance of irrigation management on plant Cu for each part (*p<0.05, **p<0.01, ***p<0.001). Error bars show the standard deviation (n=3).


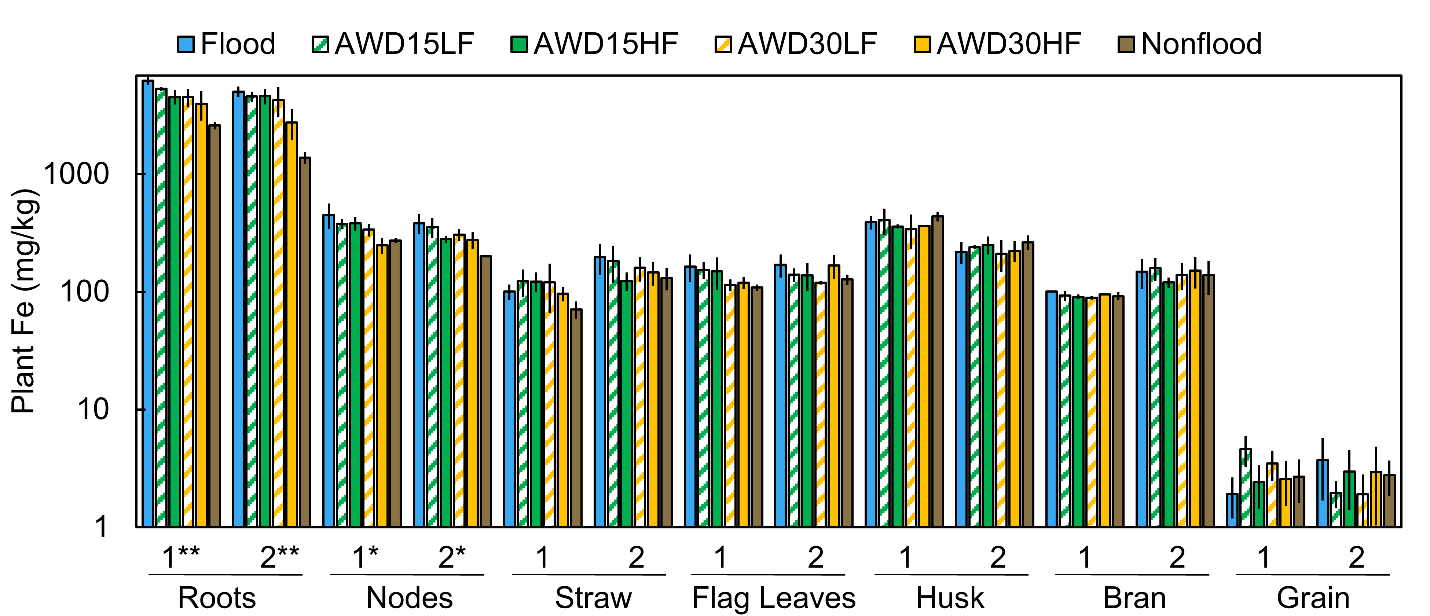


Figure S13. Effect of irrigation management on Fe concentrations in various plant parts. The number of asterisks for each year denote the significance of irrigation management on plant Fe for each part (*p<0.05, **p<0.01, ***p<0.001). Error bars show the standard deviation (n=3).


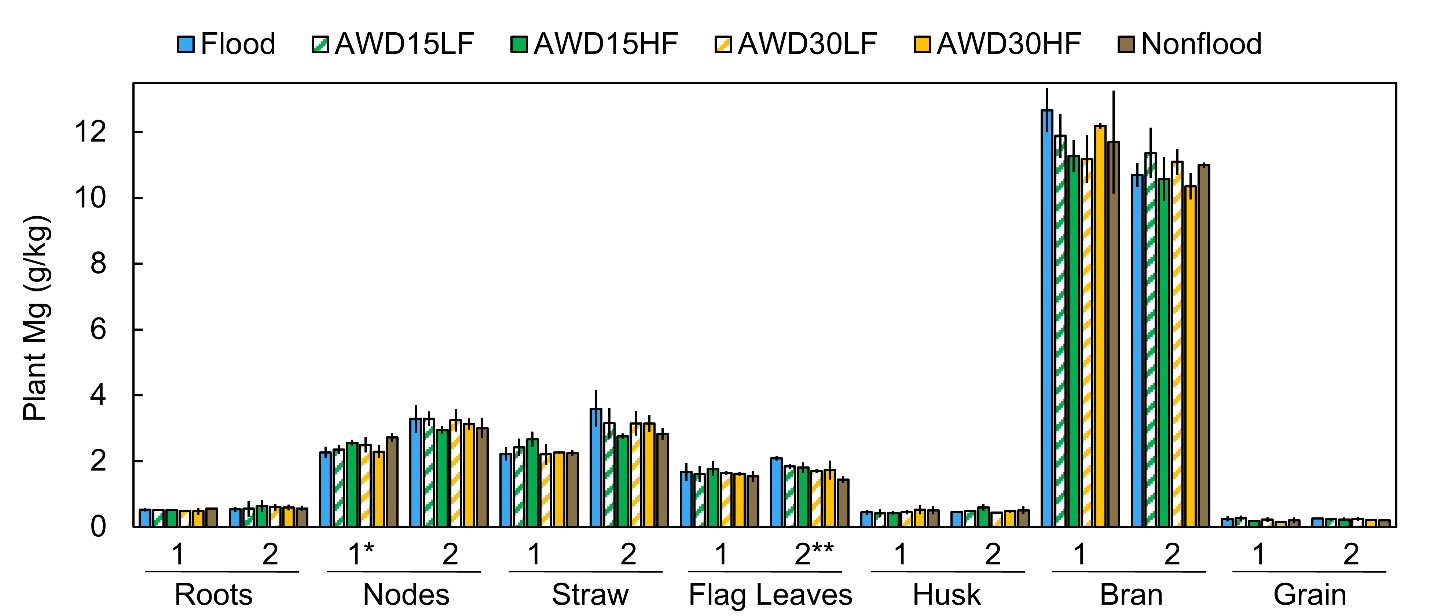


Figure S14. Effect of irrigation management on Mg concentrations in various plant parts. The number of asterisks for each year denote the significance of irrigation management on plant Mg for each part (*p<0.05, **p<0.01, ***p<0.001). Error bars show the standard deviation (n=3).


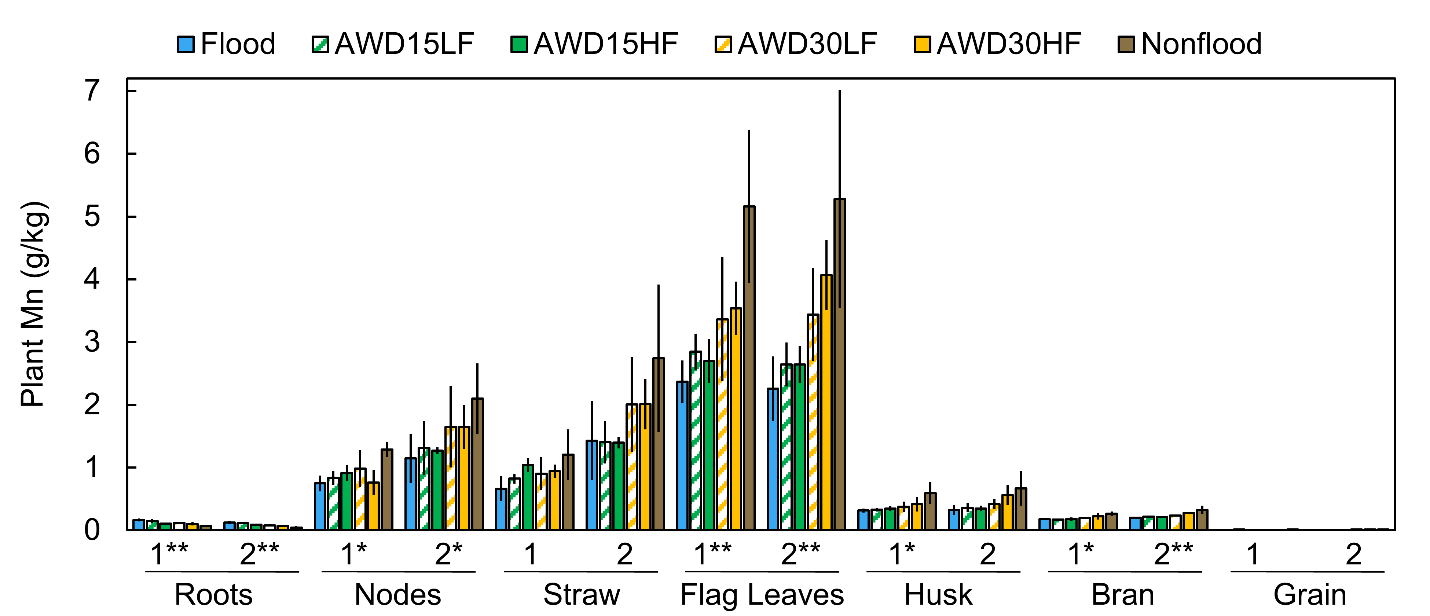


Figure S15. Effect of irrigation management on Mn concentrations in various plant parts. The number of asterisks for each year denote the significance of irrigation management on plant Mn for each part (*p<0.05, **p<0.01, ***p<0.001). Error bars show the standard deviation (n=3).


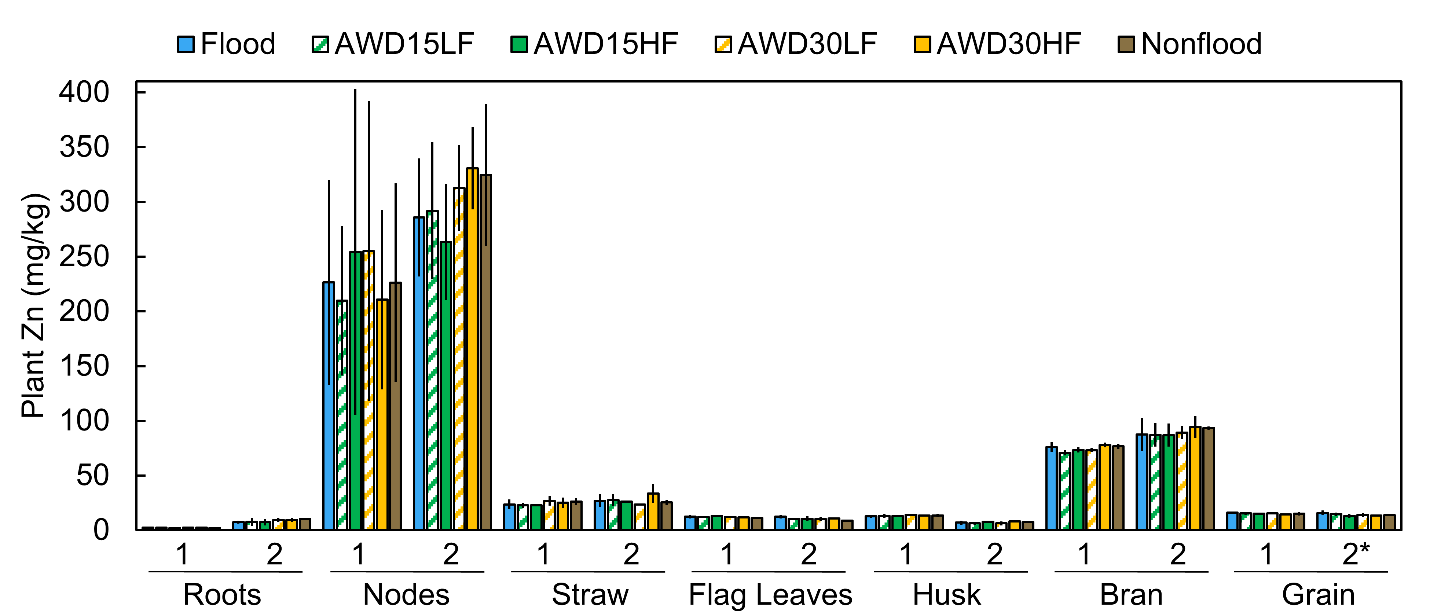


Figure S16. Effect of irrigation management on Zn concentrations in various plant parts. The number of asterisks for each year denote the significance of irrigation management on plant Zn for each part (*p<0.05, **p<0.01, ***p<0.001). Error bars show the standard deviation (n=3).


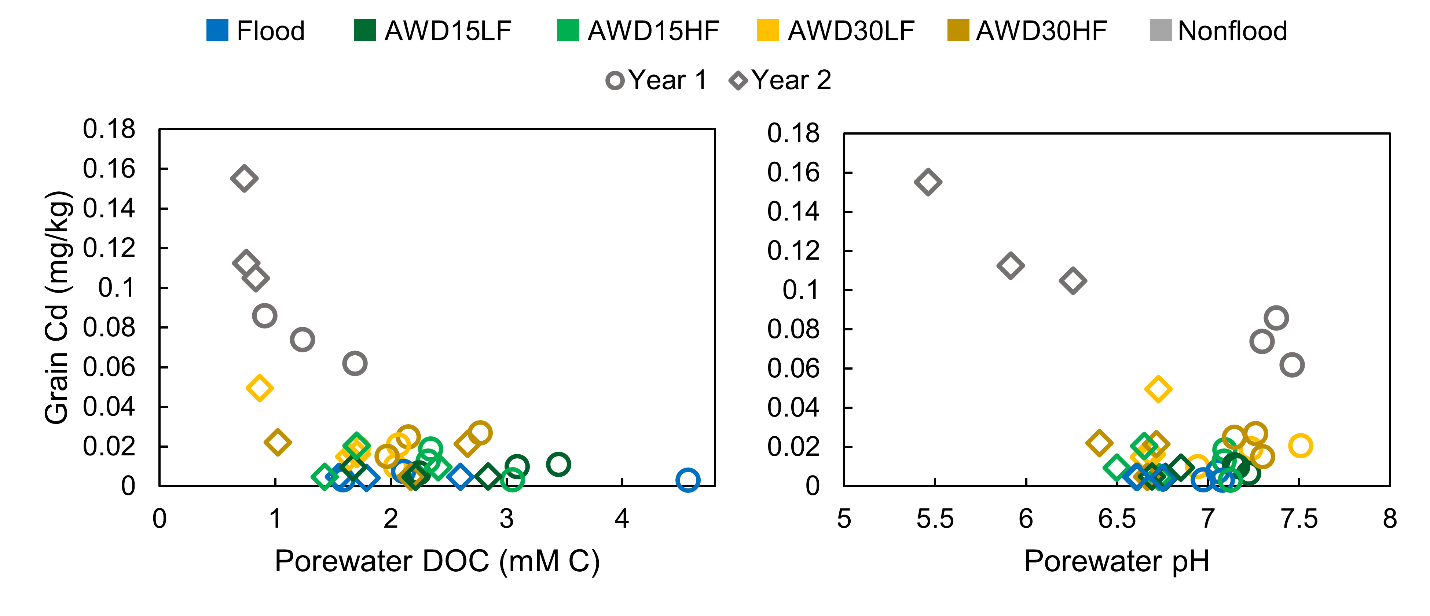


Figure S17. Correlations between grain Cd and porewater DOC and porewater pH across all irrigation managements and both years. Porewater values were averaged across the rice reproductive period.

# Supplemental Tables

Table S1. Soil elemental composition (mg/kg) as measured by total extraction (EPA 3051) and plant available nutrients (Mehlich-3).

| Element | EPA 3051 | Mehlich-3 |
| --- | --- | --- |
| As | 5.4 |  |
| Cd | 0.072 |  |
| Fe | 10,150 | 269 |
| Mn | 173 | 93 |
| Ca | 615 | 743 |
| Cu | 7.1 | 2.5 |
| K | 845 | 44 |
| Mg | 1,278 | 167 |
| P | 297 | 26 |
| S | 24 | 26 |
| Zn | 19 | 3.3 |

Table S2. Analysis of certified reference materials (mg/kg). For elemental analysis, n=12-14. For As speciation analysis n=10. Measured values are given as average (standard deviation). Reference values are given as reference value (uncertainty).

| Element | Rice Flour | Oil Palm | Spinach Leaves | Rice Flour | Oil Palm | Spinach Leaves |
| --- | --- | --- | --- | --- | --- | --- |
|  | Measured Values | | | Reference Values | | |
| As | 0.32 (0.03) |  |  | 0.29 (0.03) | |  |
| As_i_ | 0.104 (0.010) |  |  | 0.092 (0.01) | |  |
| DMA | 0.172 (0.011) |  |  | 0.18 (0.012) | |  |
| MMA | 0.013 (0.001) |  |  | 0.0116 (0.0035) | |  |
| Cd | 0.02 (0.01) | 0.04 (0.02) | 3.05 (0.73) | 0.02 (0.002) | 0.04 (0.01) | 2.88 (0.06) |
| Si |  | 17000 (1600) | |  | 20400 |  |
| Ca | 122 (11) | 7090 (587) | 16200 (2600) | 118 (6) | 6680 (509) | 15260 (660) |
| Cu | 2.2 (0.2) | 5.6 (0.3) | 12 (0) | 2.4 (0.3) | 5.6 (1) | 12 (1) |
| Fe | 8.4 (4.2) | 77 (11) | 224 (29) | 7.4 (0.9) | 80 (8) | 0 (0) |
| K | 1220 (117) | 10900 (945) | 31000 (2560) | 1280 (8) | 9770 (683) | 29000 (260) |
| Mg | 505 (32) | 3050 (274) | 9210 (861) | 560 (20) | 2730 (205) | 9000 (0) |
| Mn | 14 (7) | 405 (24) | 60 (16) | 20 (1.6) | 417 (36) | 76 (1.2) |
| Na |  |  | 18500 (1590) | |  | 18210 (230) |
| P | 1530 (118) | 1660 (157) | 5480 (377) | 1530 (80) | 1580 (103) | 5187 (67) |
| S | 1140 (86) | 1900 (83) | 5130 (342) | 1200 (20) | 1770 (146) | 5000 (0) |
| Zn | 14 (4) |  | 60 (10) | 19 (1) |  | 82 (4) |

Table S3. Plant straw biomass and rough rice yield (kg/ha). Values are shown as average (standard deviation), n=3. No comparisons were statistically significant.

|  | **Flood** | **AWD15LF** | **AWD15HF** | **AWD30LF** | **AWD30HF** | **Nonflood** |
| --- | --- | --- | --- | --- | --- | --- |
| Year 1 | | | | | | |
| **Straw** | 5390 (226) | 5520 (424) | 5760 (646) | 5320 (855) | 5010 (412) | 4650 (88) |
| **Rough Rice** | 7530 (1050) | 7540 (441) | 7260 (597) | 7460 (300) | 7280 (541) | 6400 (600) |
| Year 2 | | | | | | |
| **Straw** | 7020 (396) | 6810 (982) | 7170 (377) | 7000 (184) | 6890 (227) | 6550 (819) |
| **Rough Rice** | 7190 (392) | 6700 (234) | 6790 (361) | 6580 (363) | 6940 (50) | 6400 (538) |

Table S4. Average values for constituents measured during each year of the experiment. Porewater values are averages during the reproductive period in units of μM for elements (except nM for As and As species), mV for E_H_, and mM C for DOC. Methane is the cumulative methane emitted during the growing season (mmol CH_4_/m^2^). Values sharing the same letter were not statistically different for each constituent within each year. Values without letters were not significantly affected by irrigation management.

|  | Year 1 | | | | | | | Year 2 | | | | | | |
| --- | --- | --- | --- | --- | --- | --- | --- | --- | --- | --- | --- | --- | --- | --- |
|  | *p* | Treatment | | | | | | *p* | Treatment | | | | | |
|  |  | F | AWD 15LF | AWD 15HF | AWD 30LF | AWD 30HF | NF |  | F | AWD 15LF | AWD 15HF | AWD 30LF | AWD 30HF | NF |
| E_H_ | 0.0001 | 71^c^ | 127^bc^ | 139^bc^ | 165^b^ | 170^b^ | 242^a^ | <0.0001 | 65^c^ | 90^c^ | 87^c^ | 150^b^ | 154^b^ | 204^a^ |
| pH | 0.030 | 7.0^b^ | 7.2^ab^ | 7.1^ab^ | 7.2^ab^ | 7.2^ab^ | 7.4^a^ | 0.0012 | 6.7^a^ | 6.8^a^ | 6.6^a^ | 6.6^a^ | 6.7^a^ | 5.9^b^ |
| Mn | 0.0003 | 373^a^ | 375^a^ | 388^a^ | 347^a^ | 342^a^ | 79^b^ | 0.0014 | 306^a^ | 284^a^ | 304^a^ | 237^a^ | 221^a^ | 31^b^ |
| Fe(II) | 0.0003 | 1220^a^ | 930^ab^ | 924^ab^ | 666^b^ | 590^bc^ | 54^c^ | 0.0002 | 874^a^ | 509^ab^ | 509^ab^ | 274^bc^ | 195^bc^ | 11^c^ |
| Fe | 0.0003 | 1740^a^ | 1450^a^ | 1350^a^ | 1020^a^ | 979^a^ | 103^b^ | 0.0001 | 1170^a^ | 784^ab^ | 742^abc^ | 383^bcd^ | 240^cd^ | 45^d^ |
| As | 0.0002 | 199^a^ | 176^ab^ | 163^ab^ | 109^b^ | 111^b^ | 25^c^ | 0.0003 | 148^a^ | 125^ab^ | 102^abc^ | 57^bcd^ | 38^cd^ | 11^d^ |
| As_i_ | 0.005 | 166^a^ | 127^ab^ | 116^ab^ | 68^b^ | 67^b^ | 39^b^ | 0.0002 | 83^a^ | 65^a^ | 62^a^ | 25^b^ | 23^b^ | 17^b^ |
| As_o_ | 0.079 | 12 | 12 | 7.3 | 6.3 | 7.0 | 5.8 | 0.043 | 6.5^a^ | 6.1^a^ | 5.9^a^ | 2.4^a^ | 2.6^a^ | 1.6^a^ |
| S | 0.0004 | 31^b^ | 43^b^ | 64^b^ | 72^b^ | 87^b^ | 279^a^ | 0.0028 | 22^c^ | 93^bc^ | 107^bc^ | 144^abc^ | 224^ab^ | 303^a^ |
| P | 0.13 | 1.2 | 1.3 | 1.2 | 1.0 | 1.0 | 0.8 | 0.60 | 1.5 | 2.0 | 1.4 | 1.7 | 1.5 | 1.2 |
| K | 0.099 | 70 | 46 | 34 | 55 | 81 | 73 | 0.58 | 138 | 75 | 117 | 91 | 105 | 140 |
| Si | 0.39 | 110 | 124 | 99 | 126 | 101 | 86 | 0.43 | 78 | 74 | 93 | 76 | 77 | 67 |
| Mg | 0.013 | 1910^a^ | 2090^a^ | 1920^a^ | 1910^a^ | 1770^ab^ | 747^b^ | 0.11 | 1430 | 1530 | 1580 | 1300 | 1390 | 876 |
| Ca | 0.007 | 3240^a^ | 3590^a^ | 3290^a^ | 3280^a^ | 3130^a^ | 1420^b^ | 0.13 | 3360 | 3700 | 3890 | 3050 | 3270 | 2180 |
| DOC | 0.16 | 2.8 | 2.9 | 2.6 | 2.3 | 1.9 | 1.3 | 0.026 | 2.0^ab^ | 2.2^a^ | 1.8^ab^ | 2.0^ab^ | 1.4^ab^ | 0.77^b^ |
| CH_4_ | <.0001 | 3900^a^ | 3060^ab^ | 1920^bc^ | 1890^bc^ | 909^c^ | 379^c^ | <.0001 | 3120^a^ | 1240^b^ | 815^bc^ | 733^bc^ | 231^c^ | 88^c^ |

Table S5. Plant As concentrations (mg/kg). Values are shown as average (standard deviation), n=3. Values with different superscript letters are significantly different for each plant part.

|  | **Flood** | **AWD15LF** | **AWD15HF** | **AWD30LF** | **AWD30HF** | **Nonflood** |
| --- | --- | --- | --- | --- | --- | --- |
| Year 1 | | | | | | |
| **Root** | 66 (12)^a^ | 58 (8)^ab^ | 42 (9)^bc^ | 38 (5)^bc^ | 28 (8)^cd^ | 8 (2)^d^ |
| **Nodes** | 9.1 (1.7)^a^ | 9.2 (0.6)^a^ | 7.8 (1.2)^ab^ | 6.7 (0.2)^ab^ | 4.8 (1.5)^bc^ | 2.2 (0.1)^c^ |
| **Straw** | 2.3 (0.6)^ab^ | 2.8 (0.1)^a^ | 2.4 (0.3)^ab^ | 1.7 (0.3)^b^ | 1.3 (0.6)^bc^ | 0.4 (0.1)^c^ |
| **Flag** | 5.6 (0.2)^a^ | 5.5 (0.5)^a^ | 4.3 (0.5)^ab^ | 3.9 (0.4)^b^ | 3.8 (1.0)^b^ | 2.0 (0.1)^c^ |
| **Husk** | 0.61 (0.1)^a^ | 0.63 (0.1)^a^ | 0.52 (0.06)^ab^ | 0.43 (0.04)^bc^ | 0.4 (0.02)^bc^ | 0.31 (0.02)^c^ |
| **Bran** | 0.91 (0.04)^a^ | 0.87 (0.03)^ab^ | 0.73 (0.03)^b^ | 0.75 (0.1)^b^ | 0.71 (0.02)^bc^ | 0.55 (0.04)^c^ |
| **Ripe Grain** | 0.14 (0.015)^a^ | 0.124 (0.008)^ab^ | 0.098 (0.005)^c^ | 0.099 (0.006)^bc^ | 0.088 (0.004)^c^ | 0.073 (0.006)^c^ |
| Year 2 | | | | | | |
| **Root** | 49 (11)^a^ | 41 (5)^a^ | 42 (6)^a^ | 31 (9)^ab^ | 16 (6)^bc^ | 4 (0)^c^ |
| **Nodes** | 8.0 (0.2)^a^ | 8.0 (2.6)^a^ | 6.1 (1.0)^ab^ | 4.8 (1.9)^b^ | 3.8 (1.5)^bc^ | 1.6 (0.7)^c^ |
| **Straw** | 2.4 (0.2)^ab^ | 2.7 (0.6)^a^ | 1.8 (0.7)^abc^ | 1.4 (0.5)^abc^ | 1.0 (0.4)^bc^ | 0.7 (0.6)^c^ |
| **Flag** | 2.8 (0.4)^a^ | 2.7 (0.7)^a^ | 2.4 (0.4)^ab^ | 1.9 (0.6)^bc^ | 1.4 (0.4)^cd^ | 0.9 (0.3)^d^ |
| **Husk** | 0.41 (0.01)^a^ | 0.41 (0.04)^a^ | 0.34 (0.04)^ab^ | 0.32 (0.07)^ab^ | 0.3 (0.09)^ab^ | 0.2 (0.01)^b^ |
| **Bran** | 0.87 (0.02)^a^ | 0.84 (0.01)^ab^ | 0.74 (0.03)^b^ | 0.74 (0.07)^b^ | 0.6 (0.04)^c^ | 0.55 (0.05)^c^ |
| **Ripe Grain** | 0.124 (0.013)^a^ | 0.115 (0.015)^ab^ | 0.102 (0.009)^abc^ | 0.101 (0.009)^bc^ | 0.086 (0.011)^cd^ | 0.075 (0.012)^d^ |

Table S6. Polished grain As species (mg/kg). Values are shown as average (standard deviation), n=3. Values with different superscript letters are significantly different for each plant part.

|  | **Flood** | **AWD15LF** | **AWD15HF** | **AWD30LF** | **AWD30HF** | **Nonflood** |
| --- | --- | --- | --- | --- | --- | --- |
| Year 1 | | | | | | |
| **As_i_** | 0.122 (0.009)^a^ | 0.121 (0.005)^a^ | 0.105 (0.003)^ab^ | 0.109 (0.006)^ab^ | 0.095 (0.004)^bc^ | 0.083 (0.007)^c^ |
| **As_o_** | 0.029 (0.003)^a^ | 0.019 (0.002)^b^ | 0.015 (0.002)^bc^ | 0.012 (0.003)^c^ | 0.01 (0.001)^cd^ | 0.006 (0.001)^d^ |
| Year 2 | | | | | | |
| **As_i_** | 0.124 (0.001)^a^ | 0.114 (0.008)^ab^ | 0.111 (0.004)^b^ | 0.103 (0.003)^bc^ | 0.091 (0.005)^cd^ | 0.084 (0.002)^d^ |
| **As_o_** | 0.023 (0.003)^a^ | 0.016 (0.002)^b^ | 0.014 (0.003)^bc^ | 0.012 (0.002)^bc^ | 0.008 (0.002)^cd^ | 0.005 (0.001)^d^ |

Table S7. Plant Cd concentrations (mg/kg). Values are shown as average (standard deviation), n=3. Values with different superscript letters are significantly different for each plant part. Values without letters indicate no significant irrigation management effect.

|  | **Flood** | **AWD15LF** | **AWD15HF** | **AWD30LF** | **AWD30HF** | **Nonflood** |
| --- | --- | --- | --- | --- | --- | --- |
| Year 1 | | | | | | |
| **Root** | 0.14 (0.02)^b^ | 0.26 (0.09)^b^ | 0.22 (0.08)^b^ | 0.32 (0.12)^b^ | 0.29 (0.06)^b^ | 0.82 (0.15)^a^ |
| **Nodes** | 0.12 (0.06)^b^ | 0.29 (0.1)^b^ | 0.33 (0.14)^b^ | 0.53 (0.38)^b^ | 0.39 (0.19)^b^ | 1.83 (0.34)^a^ |
| **Straw** | 0.018 (0.01)^b^ | 0.03 (0.018)^b^ | 0.026 (0.015)^b^ | 0.068 (0.05)^b^ | 0.054 (0.042)^b^ | 0.29 (0.102)^a^ |
| **Bran** | 0.019 (0.006)^b^ | 0.034 (0.009)^b^ | 0.045 (0.024)^b^ | 0.07 (0.024)^b^ | 0.059 (0.027)^b^ | 0.24 (0.033)^a^ |
| **Ripe Grain** | 0.0047 (0.0025)^c^ | 0.0093 (0.0022)^bc^ | 0.012 (0.0078)^bc^ | 0.022 (0.0064)^b^ | 0.017 (0.0058)^bc^ | 0.074 (0.0119)^a^ |
| Year 2 | | | | | | |
| **Root** | 0.11 (0.05)^c^ | 0.15 (0.01)^bc^ | 0.18 (0.08)^bc^ | 0.26 (0.04)^bc^ | 0.38 (0.24)^b^ | 1.03 (0.03)^a^ |
| **Nodes** | 0.13 (0.09)^b^ | 0.28 (0.11)^b^ | 0.58 (0.42)^b^ | 0.7 (0.25)^b^ | 0.97 (0.57)^b^ | 3.25 (0.59)^a^ |
| **Straw** | 0.021 (0.001) | 0.022 (0.000) | 0.042 (0.029) | 0.032 (0.021) | 0.079 (0.055) | 0.28 (0.23) |
| **Bran** | 0.006 (0.000)^b^ | 0.01 (0.007)^b^ | 0.019 (0.015)^b^ | 0.028 (0.019)^b^ | 0.054 (0.036)^b^ | 0.20 (0.046)^a^ |
| **Ripe Grain** | 0.0046 (0.0003)^b^ | 0.0064 (0.0027)^b^ | 0.012 (0.0081)^b^ | 0.016 (0.0098)^b^ | 0.027 (0.020)^b^ | 0.12 (0.027)^a^ |

Table S8. Plant P concentrations (g/kg). Values are shown as average (standard deviation), n=3. Values with different superscript letters are significantly different for each plant part. Values without letters indicate no significant irrigation management effect.

|  | **Flood** | **AWD15LF** | **AWD15HF** | **AWD30LF** | **AWD30HF** | **Nonflood** |
| --- | --- | --- | --- | --- | --- | --- |
| Year 1 | | | | | | |
| **Root** | 0.25 (0.01)^a^ | 0.27 (0.03)^a^ | 0.25 (0.02)^a^ | 0.26 (0.02)^a^ | 0.23 (0.02)^ab^ | 0.19 (0.02)^b^ |
| **Nodes** | 1.4 (0.3)^a^ | 1.2 (0.1)^a^ | 1.4 (0.2)^a^ | 1.3 (0.2)^a^ | 1.0 (0.0)^a^ | 1.0 (0.0)^a^ |
| **Straw** | 1.1 (0.2)^ab^ | 1.2 (0.2)^ab^ | 1.4 (0.2)^a^ | 1.2 (0.3)^ab^ | 1.1 (0.1)^ab^ | 0.8 (0.2)^b^ |
| **Flag** | 1.7 (0.2) | 1.6 (0.0) | 1.9 (0.2) | 1.7 (0.2) | 1.6 (0.0) | 1.4 (0.2) |
| **Husk** | 0.63 (0.17) | 0.55 (0.25) | 0.57 (0.07) | 0.69 (0.23) | 0.81 (0.42) | 0.73 (0.3) |
| **Bran** | 21 (1) | 20 (1) | 19 (1) | 19 (1) | 20 (0) | 20 (3) |
| **Ripe Grain** | 1.1 (0.2) | 1.1 (0.2) | 0.9 (0.1) | 1.0 (0.2) | 0.8 (0) | 1.0 (0.3) |
| Year 2 | | | | | | |
| **Root** | 0.29 (0.14) | 0.2 (0.01) | 0.21 (0.01) | 0.21 (0.00) | 0.21 (0.00) | 0.19 (0.01) |
| **Nodes** | 2.7 (0.3)^a^ | 2.6 (0.3)^a^ | 2.2 (0.2)^ab^ | 2.5 (0.1)^ab^ | 2.5 (0.2)^a^ | 1.7 (0.4)^b^ |
| **Straw** | 2.0 (0.2) | 2.0 (0.3) | 1.8 (0.2) | 1.8 (0.2) | 1.7 (0.1) | 1.4 (0.3) |
| **Flag** | 2.2 (0.1)^a^ | 2.0 (0.1)^ab^ | 2.0 (0.2)^ab^ | 2.0 (0.0)^ab^ | 2.0 (0.1)^ab^ | 1.7 (0.1)^b^ |
| **Husk** | 0.55 (0.04) | 0.58 (0.04) | 0.87 (0.26) | 0.54 (0.03) | 0.64 (0.12) | 0.74 (0.33) |
| **Bran** | 24 (1) | 26 (1) | 24 (1) | 25 (0) | 24 (1) | 25 (1) |
| **Ripe Grain** | 1.3 (0.1) | 1.2 (0.1) | 1.2 (0.2) | 1.3 (0.2) | 1.2 (0.0) | 1.2 (0.0) |

Table S9. Plant K concentrations (g/kg). Values are shown as average (standard deviation), n=3. Values with different superscript letters are significantly different for each plant part. Values without letters indicate no significant irrigation management effect.

|  | **Flood** | **AWD15LF** | **AWD15HF** | **AWD30LF** | **AWD30HF** | **Nonflood** |
| --- | --- | --- | --- | --- | --- | --- |
| Year 1 | | | | | | |
| **Root** | 0.6 (0.1)^ab^ | 0.6 (0.1)^ab^ | 0.6 (0.1)^ab^ | 0.5 (0.0)^b^ | 0.5 (0.2)^b^ | 0.9 (0.1)^a^ |
| **Nodes** | 44 (5) | 46 (3) | 44 (4) | 46 (10) | 39 (1) | 39 (0) |
| **Straw** | 33 (8) | 28 (4) | 24 (1) | 31 (3) | 29 (6) | 31 (5) |
| **Flag** | 13 (1)^a^ | 12 (1)^a^ | 13 (1)^ab^ | 12 (1)^ab^ | 12 (0)^ab^ | 11 (0)^b^ |
| **Husk** | 4.5 (0.1)^a^ | 4.5 (0.3)^a^ | 4.4 (0.2)^a^ | 4.1 (0.1)^a^ | 4.1 (0.4)^a^ | 3.9 (0.1)^a^ |
| **Bran** | 18 (1) | 17 (1) | 16 (1) | 16 (1) | 17 (1) | 17 (3) |
| **Ripe Grain** | 0.87 (0.13) | 0.9 (0.07) | 0.76 (0.05) | 0.81 (0.06) | 0.76 (0.07) | 0.86 (0.13) |
| Year 2 | | | | | | |
| **Root** | 0.5 (0) | 0.6 (0.3) | 0.7 (0.2) | 0.7 (0.1) | 0.7 (0.1) | 0.9 (0) |
| **Nodes** | 45 (2)^a^ | 45 (2)^a^ | 40 (2)^b^ | 42 (1)^ab^ | 41 (1)^ab^ | 41 (2)^ab^ |
| **Straw** | 28 (2) | 28 (4) | 30 (2) | 26 (3) | 30 (1) | 27 (2) |
| **Flag** | 13 (1)^a^ | 12 (0)^ab^ | 12 (0)^ab^ | 12 (0)^b^ | 12 (1)^ab^ | 11 (1)^b^ |
| **Husk** | 4.4 (0.1) | 4.3 (0.0) | 4.2 (0.2) | 4.2 (0.1) | 4.1 (0.2) | 3.9 (0.3) |
| **Bran** | 18 (2) | 19 (2) | 18 (2) | 19 (2) | 18 (1) | 20 (0) |
| **Ripe Grain** | 0.81 (0.10) | 0.81 (0.09) | 0.81 (0.16) | 0.82 (0.14) | 0.79 (0.04) | 0.79 (0.04) |

Table S10. Plant Si concentrations (g/kg). Values are shown as average (standard deviation), n=3. No significant irrigation management effects were observed.

|  | **Flood** | **AWD15LF** | **AWD15HF** | **AWD30LF** | **AWD30HF** | **Nonflood** |
| --- | --- | --- | --- | --- | --- | --- |
| Year 1 | | | | | | |
| **Root** | 7.8 (1.0) | 7.9 (1.2) | 7.4 (0.7) | 6.8 (1) | 6.4 (0.8) | 8.3 (1.5) |
| **Nodes** | 17 (3) | 17 (2) | 17 (3) | 18 (3) | 17 (1) | 17 (2) |
| **Straw** | 20 (4) | 21 (2) | 21 (4) | 20 (3) | 19 (0) | 20 (2) |
| **Flag** | 42 (11) | 39 (6) | 36 (8) | 36 (8) | 40 (7) | 40 (7) |
| **Husk** | 51 (9) | 50 (7) | 47 (8) | 47 (5) | 44 (7) | 46 (10) |
| Year 2 | | | | | | |
| **Root** | 6.0 (0.5) | 6.1 (2.2) | 7.4 (3) | 6.8 (0.5) | 5.6 (0.2) | 5.0 (0.7) |
| **Nodes** | 14 (3) | 16 (5) | 18 (5) | 15 (3) | 13 (3) | 13 (2) |
| **Straw** | 18 (2) | 19 (5) | 16 (3) | 19 (3) | 16 (3) | 18 (5) |
| **Flag** | 27 (4) | 28 (9) | 29 (10) | 26 (6) | 24 (6) | 23 (6) |
| **Husk** | 45 (4) | 50 (6) | 48 (6) | 51 (4) | 42 (6) | 39 (4) |

Table S11. Plant Cu concentrations (mg/kg). Values are shown as average (standard deviation), n=3. Values with different superscript letters are significantly different for each plant part. Values without letters indicate no significant irrigation management effect.

|  | **Flood** | **AWD15LF** | **AWD15HF** | **AWD30LF** | **AWD30HF** | **Nonflood** |
| --- | --- | --- | --- | --- | --- | --- |
| Year 1 | | | | | | |
| **Root** | 2.1 (0.1)^b^ | 3.2 (1.2)^b^ | 3.4 (0.9)^b^ | 4.3 (0.4)^ab^ | 3.8 (0.3)^b^ | 6.8 (1.6)^a^ |
| **Nodes** | 1.1 (0.7)^c^ | 2.1 (1.4)^bc^ | 4.2 (1.2)^bc^ | 5.4 (1.8)^bc^ | 5.7 (1.9)^b^ | 16.7 (1.9)^a^ |
| **Straw** | 0.24 (0.01)^b^ | 0.31 (0.14)^ab^ | 1.04 (1.14)^b^ | 0.5 (0.35)^b^ | 1.01 (0.27)^b^ | 2.49 (0.25)^a^ |
| **Flag** | 2.4 (0.7)^c^ | 2.6 (0.7)^bc^ | 3.8 (0.7)^ab^ | 3.2 (0.2)^bc^ | 3.4 (0.4)^bc^ | 4.8 (0.5)^a^ |
| **Husk** | 1.4 (0.3)^b^ | 0.8^b^ | 1.6 (0.4)^b^ | 1.5 (0.2)^b^ | 1.7 (0.2)^ab^ | 2.8 (0.7)^a^ |
| **Bran** | 5.4 (0.8)^b^ | 5.1 (0.5)^b^ | 7.8 (2.9)^ab^ | 6.8 (1.2)^ab^ | 9.6 (2.1)^ab^ | 10.8 (1.6)^a^ |
| **Ripe Grain** | 1.1 (0.2)^c^ | 1.1 (0.1)^bc^ | 1.5 (0.1)^bc^ | 1.7 (0.3)^bc^ | 1.8 (0.2)^b^ | 2.7 (0.4)^a^ |
| Year 2 | | | | | | |
| **Root** | 5.5 (1.3)^b^ | 6.5 (3.2)^ab^ | 7.2 (0.9)^ab^ | 8.2 (2.4)^ab^ | 13.2 (5.7)^ab^ | 14.8 (3.0)^a^ |
| **Nodes** | 6.5 (6.0)^b^ | 7.9 (4.5)^b^ | 7.3 (1.2)^b^ | 12.5 (7.1)^ab^ | 15.7 (6.6)^ab^ | 21.8 (3.8)^a^ |
| **Straw** | 3.3 (1.8) | 3.6 (0.72) | 3.27 (0.41) | 4.26 (1.8) | 4.27 (1.7) | 5.9 (2.4) |
| **Flag** | 5.2 (1.4)^b^ | 4.9 (0.9)^b^ | 5.6 (1.3)^ab^ | 5.9 (1.1)^ab^ | 6.9 (1.0)^ab^ | 7.5 (0.5)^a^ |
| **Husk** | 2.4 (0.3)^b^ | 2.4 (0.6)^b^ | 2.6 (0.4)^b^ | 2.4 (0.5)^b^ | 3.1 (0.5)^ab^ | 3.8 (0.3)^a^ |
| **Bran** | 7.7 (1.9)^c^ | 9 (1.4)^bc^ | 8.5 (0.3)^c^ | 10.2 (2.3)^bc^ | 12 (2.1)^b^ | 16.5 (1.2)^a^ |
| **Ripe Grain** | 2.3 (1.1)^b^ | 2.5 (0.7)^b^ | 2.4 (0.4)^b^ | 3.0 (1.1)^b^ | 3.4 (0.8)^ab^ | 4.6 (0.4)^a^ |

Table S12. Plant Fe concentrations (mg/kg). Values are shown as average (standard deviation), n=3. Values with different superscript letters are significantly different for each plant part. Values without letters indicate no significant irrigation management effect.

|  | **Flood** | **AWD15LF** | **AWD15HF** | **AWD30LF** | **AWD30HF** | **Nonflood** |
| --- | --- | --- | --- | --- | --- | --- |
| Year 1 | | | | | | |
| **Root** | 6200 (508)^a^ | 5310 (211)^ab^ | 4530 (637)^abc^ | 4500 (823)^abc^ | 3970 (1120)^bc^ | 2590 (202)^c^ |
| **Nodes** | 451 (111)^a^ | 377 (37)^ab^ | 382 (51)^ab^ | 338 (43)^ab^ | 248 (37)^b^ | 273 (12)^b^ |
| **Straw** | 100 (15) | 123 (33) | 123 (23) | 120 (54) | 96 (14) | 71 (12) |
| **Flag** | 164 (44) | 153 (25) | 150 (46) | 114 (13) | 120 (15) | 109 (7) |
| **Husk** | 390 (51) | 408 (102) | 360 (14) | 342 (113) | 363 (8) | 437 (42) |
| **Bran** | 101 (2) | 93 (9) | 91 (4) | 89 (3) | 95 (0) | 92 (7) |
| **Ripe Grain** | 1.9 (0.7) | 4.6 (1.3) | 2.4 (1) | 3.5 (1) | 2.6 (1.1) | 2.7 (1.1) |
| Year 2 | | | | | | |
| **Root** | 5030 (523)^a^ | 4570 (460)^ab^ | 4590 (698)^ab^ | 4260 (1230)^ab^ | 2750 (807)^bc^ | 1380 (158)^c^ |
| **Nodes** | 383 (73)^a^ | 356 (71)^a^ | 280 (18)^ab^ | 306 (34)^ab^ | 276 (46)^ab^ | 201 (4)^b^ |
| **Straw** | 197 (57) | 183 (64) | 124 (23) | 161 (38) | 146 (33) | 132 (28) |
| **Flag** | 170 (38) | 140 (19) | 138 (37) | 120 (5) | 167 (39) | 128 (13) |
| **Husk** | 219 (47) | 240 (9) | 251 (43) | 211 (64) | 224 (45) | 264 (37) |
| **Bran** | 148 (42) | 160 (36) | 120 (12) | 139 (36) | 152 (46) | 139 (44) |
| **Ripe Grain** | 3.7 (2) | 2 (0.5) | 3 (1.6) | 1.9 (0.9) | 2.9 (1.9) | 2.8 (0.9) |

Table S13. Plant Mg concentrations (g/kg). Values are shown as average (standard deviation), n=3. Values with different superscript letters are significantly different for each plant part. Values without letters indicate no significant irrigation management effect.

|  | **Flood** | **AWD15LF** | **AWD15HF** | **AWD30LF** | **AWD30HF** | **Nonflood** |
| --- | --- | --- | --- | --- | --- | --- |
| Year 1 | | | | | | |
| **Root** | 0.53 (0.06) | 0.51 (0.02) | 0.52 (0.02) | 0.49 (0.03) | 0.48 (0.11) | 0.56 (0.01) |
| **Nodes** | 2.3 (0.2)^a^ | 2.4 (0.1)^a^ | 2.6 (0.1)^a^ | 2.5 (0.2)^a^ | 2.3 (0.2)^a^ | 2.7 (0.1)^a^ |
| **Straw** | 2.2 (0.2) | 2.4 (0.3) | 2.7 (0.2) | 2.2 (0.3) | 2.3 (0) | 2.2 (0.1) |
| **Flag** | 1.7 (0.3) | 1.6 (0.2) | 1.8 (0.2) | 1.6 (0.1) | 1.6 (0.1) | 1.5 (0.2) |
| **Husk** | 0.45 (0.07) | 0.41 (0.12) | 0.43 (0.06) | 0.45 (0.07) | 0.52 (0.14) | 0.5 (0.12) |
| **Bran** | 13 (1) | 12 (1) | 11 (0) | 11 (1) | 12 (0) | 12 (2) |
| **Ripe Grain** | 0.25 (0.08) | 0.27 (0.08) | 0.19 (0.02) | 0.22 (0.07) | 0.15 (0.01) | 0.21 (0.08) |
| Year 2 | | | | | | |
| **Root** | 0.54 (0.07) | 0.56 (0.24) | 0.63 (0.19) | 0.60 (0.10) | 0.60 (0.09) | 0.56 (0.09) |
| **Nodes** | 3.3 (0.4) | 3.3 (0.2) | 2.9 (0.1) | 3.2 (0.3) | 3.1 (0.2) | 3.0 (0.3) |
| **Straw** | 3.6 (0.6) | 3.2 (0.5) | 2.8 (0.1) | 3.1 (0.4) | 3.1 (0.3) | 2.8 (0.2) |
| **Flag** | 2.1 (0.1)^a^ | 1.8 (0.1)^ab^ | 1.8 (0.2)^ab^ | 1.7 (0.1)^ab^ | 1.7 (0.3)^ab^ | 1.4 (0.1)^b^ |
| **Husk** | 0.46 (0.01) | 0.48 (0.01) | 0.59 (0.11) | 0.44 (0.01) | 0.48 (0.05) | 0.51 (0.12) |
| **Bran** | 11 (0) | 11 (1) | 11 (1) | 11 (0) | 10 (0) | 11 (0) |
| **Ripe Grain** | 0.26 (0.03) | 0.24 (0.04) | 0.23 (0.06) | 0.25 (0.06) | 0.21 (0.00) | 0.21 (0.00) |

Table S14. Plant Mn concentrations (g/kg). Values are shown as average (standard deviation), n=3. Values with different superscript letters are significantly different for each plant part. Values without letters indicate no significant irrigation management effect.

|  | **Flood** | **AWD15LF** | **AWD15HF** | **AWD30LF** | **AWD30HF** | **Nonflood** |
| --- | --- | --- | --- | --- | --- | --- |
| Year 1 | | | | | | |
| **Root** | 0.17 (0.02)^a^ | 0.15 (0.03)^ab^ | 0.11 (0.01)^bc^ | 0.12 (0.01)^abc^ | 0.1 (0.03)^bc^ | 0.07 (0.00)^c^ |
| **Nodes** | 0.75 (0.12)^b^ | 0.84 (0.11)^ab^ | 0.91 (0.12)^ab^ | 0.98 (0.3)^ab^ | 0.76 (0.20)^b^ | 1.29 (0.12)^a^ |
| **Straw** | 0.66 (0.20) | 0.82 (0.08) | 1.04 (0.11) | 0.9 (0.26) | 0.94 (0.11) | 1.21 (0.4) |
| **Flag** | 2.4 (0.3)^b^ | 2.8 (0.3)^b^ | 2.7 (0.4)^b^ | 3.4 (1.0)^ab^ | 3.5 (0.4)^ab^ | 5.2 (1.2)^a^ |
| **Husk** | 0.32 (0.03)^b^ | 0.33 (0.03)^ab^ | 0.35 (0.04)^ab^ | 0.37 (0.08)^ab^ | 0.42 (0.12)^ab^ | 0.6 (0.17)^a^ |
| **Bran** | 0.18 (0.02)^b^ | 0.17 (0.01)^b^ | 0.18 (0.03)^b^ | 0.2 (0.02)^ab^ | 0.23 (0.05)^ab^ | 0.26 (0.03)^a^ |
| **Ripe Grain** | 0.011 (0.001) | 0.011 (0.002) | 0.01 (0.001) | 0.011 (0.002) | 0.01 (0.001) | 0.013 (0.004) |
| Year 2 | | | | | | |
| **Root** | 0.12 (0.02)^a^ | 0.12 (0.01)^a^ | 0.09 (0.01)^ab^ | 0.08 (0.02)^ab^ | 0.07 (0.02)^ab^ | 0.04 (0.03)^b^ |
| **Nodes** | 1.15 (0.39)^b^ | 1.31 (0.43)^ab^ | 1.27 (0.05)^ab^ | 1.65 (0.65)^ab^ | 1.65 (0.35)^ab^ | 2.1 (0.56)^a^ |
| **Straw** | 1.43 (0.63) | 1.4 (0.34) | 1.4 (0.09) | 2.01 (0.76) | 2.01 (0.4) | 2.74 (1.18) |
| **Flag** | 2.3 (0.5)^b^ | 2.6 (0.4)^b^ | 2.6 (0.3)^b^ | 3.4 (0.7)^ab^ | 4.1 (0.6)^ab^ | 5.3 (1.7)^a^ |
| **Husk** | 0.33 (0.08) | 0.36 (0.07) | 0.35 (0.04) | 0.42 (0.08) | 0.57 (0.16) | 0.67 (0.28) |
| **Bran** | 0.20 (0.02)^b^ | 0.22 (0.02)^b^ | 0.21 (0.01)^b^ | 0.24 (0.02)^b^ | 0.28 (0.02)^ab^ | 0.33 (0.07)^a^ |
| **Ripe Grain** | 0.011 (0.002) | 0.011 (0.002) | 0.01 (0.001) | 0.012 (0.002) | 0.012 (0.001) | 0.013 (0.001) |

Table S15. Plant Zn concentrations (mg/kg). Values are shown as average (standard deviation), n=3. Values with different superscript letters are significantly different for each plant part. Values without letters indicate no significant irrigation management effect.

|  | **Flood** | **AWD15LF** | **AWD15HF** | **AWD30LF** | **AWD30HF** | **Nonflood** |
| --- | --- | --- | --- | --- | --- | --- |
| Year 1 | | | | | | |
| **Root** | 2.4 (0.0) | 2.4 (0.2) | 2.2 (0.3) | 2.3 (0.1) | 2.4 (0.2) | 2.1 (0.2) |
| **Nodes** | 226 (94) | 210 (68) | 254 (149) | 255 (137) | 211 (82) | 226 (91) |
| **Straw** | 24 (5) | 23 (2) | 23 (0) | 27 (4) | 25 (5) | 26 (3) |
| **Flag** | 13 (2) | 12 (0) | 13 (1) | 12 (0) | 12 (1) | 11 (1) |
| **Husk** | 13 (1) | 13 (2) | 13 (0) | 14 (1) | 14 (1) | 14 (1) |
| **Bran** | 76 (5) | 71 (2) | 73 (2) | 73 (2) | 78 (2) | 77 (2) |
| **Ripe Grain** | 16 (1) | 15 (1) | 15 (0) | 16 (1) | 15 (0) | 15 (1) |
| Year 2 | | | | | | |
| **Root** | 7.5 (1.5) | 7.7 (3.9) | 7.6 (3.0) | 9.5 (2.1) | 9.6 (1.7) | 10.4 (1.1) |
| **Nodes** | 286 (54) | 292 (62) | 264 (53) | 313 (39) | 331 (37) | 325 (65) |
| **Straw** | 27 (6) | 28 (5) | 26 (0) | 24 (1) | 34 (9) | 25 (2) |
| **Flag** | 12 (2) | 11 (1) | 11 (2) | 10 (2) | 11 (1) | 9 (1) |
| **Husk** | 7 (2) | 6 (2) | 8 (1) | 6 (2) | 8 (1) | 8 (0) |
| **Bran** | 88 (15) | 87 (11) | 87 (11) | 89 (6) | 95 (10) | 93 (1) |
| **Ripe Grain** | 16 (2)^a^ | 15 (2)^ab^ | 13 (2)^b^ | 14 (2)^ab^ | 14 (0)^ab^ | 14 (0)^ab^ |
